# 90% yield production of polymer nano-memristor for in-memory computing

**DOI:** 10.1038/s41467-021-22243-8

**Published:** 2021-03-31

**Authors:** Bin Zhang, Weilin Chen, Jianmin Zeng, Fei Fan, Junwei Gu, Xinhui Chen, Lin Yan, Guangjun Xie, Shuzhi Liu, Qing Yan, Seung Jae Baik, Zhi-Guo Zhang, Weihua Chen, Jie Hou, Mohamed E. El-Khouly, Zhang Zhang, Gang Liu, Yu Chen

**Affiliations:** 1 Key Laboratory for Advanced Materials and Joint International Research Laboratory of Precision Chemistry and Molecular Engineering, Feringa Nobel Prize Scientist Joint Research Center, School of Chemistry and Molecular Engineering, East China University of Science and Technology, Shanghai, China; 2 School of Electronic Information and Electrical Engineering, Shanghai Jiao Tong University, Shanghai, China; 3 School of Electronic Science and Applied Physics, Hefei University of Technology, Hefei, China; 4 School of Chemistry and Chemical Engineering, Shanghai Jiao Tong University, Shanghai, China; 5 Shaanxi Key Laboratory of Macromolecular Science and Technology, School of Chemistry and Chemical Engineering, Northwestern Polytechnical University, Xi’ an, Shaanxi China; 6 School of Electronic and Electrical Engineering, Hankyong National University, Anseong-si, Gyeonggi-do Korea; 7 Green Catalysis Center and College of Chemistry, Zhengzhou University, Zhengzhou, China; 8 Institude of Basic and Applied Sciences, Egypt-Japan University of Science and Technology (E-JUST), Alexandria, Egypt

**Keywords:** Electronic materials, Conjugated polymers, Electronic devices, Information storage

## Abstract

Polymer memristors with light weight and mechanical flexibility are preeminent candidates for low-power edge computing paradigms. However, the structural inhomogeneity of most polymers usually leads to random resistive switching characteristics, which lowers the production yield and reliability of nanoscale devices. In this contribution, we report that by adopting the two-dimensional conjugation strategy, a record high 90% production yield of polymer memristors has been achieved with miniaturization and low power potentials. By constructing coplanar macromolecules with 2D conjugated thiophene derivatives to enhance the *π*–*π* stacking and crystallinity of the thin film, homogeneous switching takes place across the entire polymer layer, with fast responses in 32 ns, D2D variation down to 3.16% ~ 8.29%, production yield approaching 90%, and scalability into 100 nm scale with tiny power consumption of ~ 10^−15^ J/bit. The polymer memristor array is capable of acting as both the arithmetic-logic element and multiply-accumulate accelerator for neuromorphic computing tasks.

## Abstract (web summary)

Though polymer memristors are promising for low‐power flexible edge computing applications, realizing efficient nanometer‐scale arrays remains a challenge. Here, the authors report a record high 90% production yield in nm‐scale 2D conjugated polymer memristors with homogeneous resistive switching.

## Introduction

Entering the nowadays Internet of Thing, Big Data, and artificial intelligence era, global data explodes exponentially in the great diverse area of municipal traffic control, domestic security surveillance, medication service, industrial production, and etc^1^, and has reached 40 trillion gigabytes in total or 5 terabytes per person by 2020^2,3^. These huge amount of analog signals gathered at the sensor terminals are usually uploaded to the Cloud for massive storage and on-demand processing via remote data center and shared computing resources. Although the relocation of the heaving-lifting data storage, crunching, and processing from the user-end devices to the virtual cyperspaces demonstrates advantages of cost-saving, improved productivity, and information security, the non-differentiated transmission of the redundant invalid data to the Cloud nevertheless leads to significant waste of the computing resources and tremendous power consumption in the meanwhile^4–6^. The energy starvation is predicted as the most severe challenge that is faced by the information technology industry at the middle of this century^7^. New electronic devices and low-power edge computing paradigm that takes place at or near the physical equipments or source of data are therefore highly desired to provide important supplements to the Cloud computing for real-time data processing and pre-screening, especially when fast responses to the surrounding happenings are necessary, e.g. instant detection and dodging of on-road obstacle during vehicle autopiloting.

The recently well studied memristors, with their CMOS compatibility^7^, fast switching speed and low power potential^8^, are considered as promising candidates for high density information storage^9^. The non-volatile reconfiguration of their resistance, together with the simple two terminal structure and three-dimensional integrating capability, also makes memristor crossbar arrays capable of executing large-scale in-memory computing tasks^10,11^. Eliminating the frequent data movement between the physically separated central processing unit and memory hierarchy in the von Neumann architectured computer system will both enhance the computation efficiency and lower the energy consumption when dealing with data-intensive workloads. In particular, the incorporation of light weight polymer as the switching matrix in memristors demonstrates superior advantages in low-power flexible edge computing applications^12–16^. Running on the mechanisms of charge trapping and detrapping, charge transfer (CT), electrochemical redox reaction, conformation reconfiguration, ion migration, and etc.^17^, localized resistive switching occurs in polymer memristor devices, wherein the highly conductive regions (or conductive filaments) are generated more easily and preferentially under the locally intensified electric field in defects-rich areas^18–21^. Due to the spatial inhomogeneity of the defects and internal electric field, and thus the random distribution of conductive filaments inside the switching matrix, downscaling of the memristors into nanometer scale may results in the allocation of certain devices in regions that show different electrical behavior or do not exhibit any resistive switching characteristics at all. Although polymer memristor shows ambitious scientific importance for low-power edge computing applications, their poor fabrication yield and reliability are still primary issues that hinder them from direct practical applications.

Molecular chemistry of organic materials offers great possibility to fine-tune the geometry and electronic structures of functional monomers and polymers through the molecular design- cum-synthesis strategy^22–24^. For instance, through manipulating the coplanarity of macromolecules with two-dimensionally (2D) conjugated electron donor moieties in the polymer skeleton, the extent of intermolecular *π*–*π* stacking and crystallinity of the organic thin films can be effectively improved^25–28^. The resultant better regioregularity may promote effective charge transport through the extended *π*-conjugation system and enhance the overall performance of polymer optoelectronic devices.

In this work, we report the design of a 2D conjugated polymer PBDTT-BQTPA that exhibits homogeneous resistive switching characteristics for ultra-miniaturization and low-power potential applications. With the incorporation of redox active triphenylamine moieties in the pendant unities, the polymer device can transit between the ON and OFF states within ~32 ns, with small programming voltages of ~±0.30 V. More importantly, the presence of the coplanar bis(thiophene)-4,8-dihydrobenzo[1,2-b:4,5-b]dithiophene (BDTT) chromophores can regulate the stacking ordering of the electron donating (D) thiophene-accepting (A) quinoxaline pairs and the crystallinity of the polymer thin film, resulting in bulk phase resistive switching phenomenon that propagates across the entire PBDTT-BQTPA layer. With the good retention exceeding 10^4^ s, endurance over 10^8^ working cycles, small device-to-device variation of 3.16–8.29% for the programming voltages and ON/OFF state resistances, and record high production yield approaching 90%, the delocalized resistive switching greatly enhances the reliability of the PBDTT-BQTPA based memristors, making downscaling of the device into 100 nanometer scale possible for low-power edge computing applications. As a demonstration, we display that both the in-memory Boolean logic and arithmetic operations for general purpose computing, as well as the binary neural network (BNN) as hardware accelerator for pattern recognition tasks, can be implemented with the present PBDTT-BQTPA based memristor devices.

## Results

### Design and synthesis of 2D conjugated memristive polymer

**Fig. 1:**
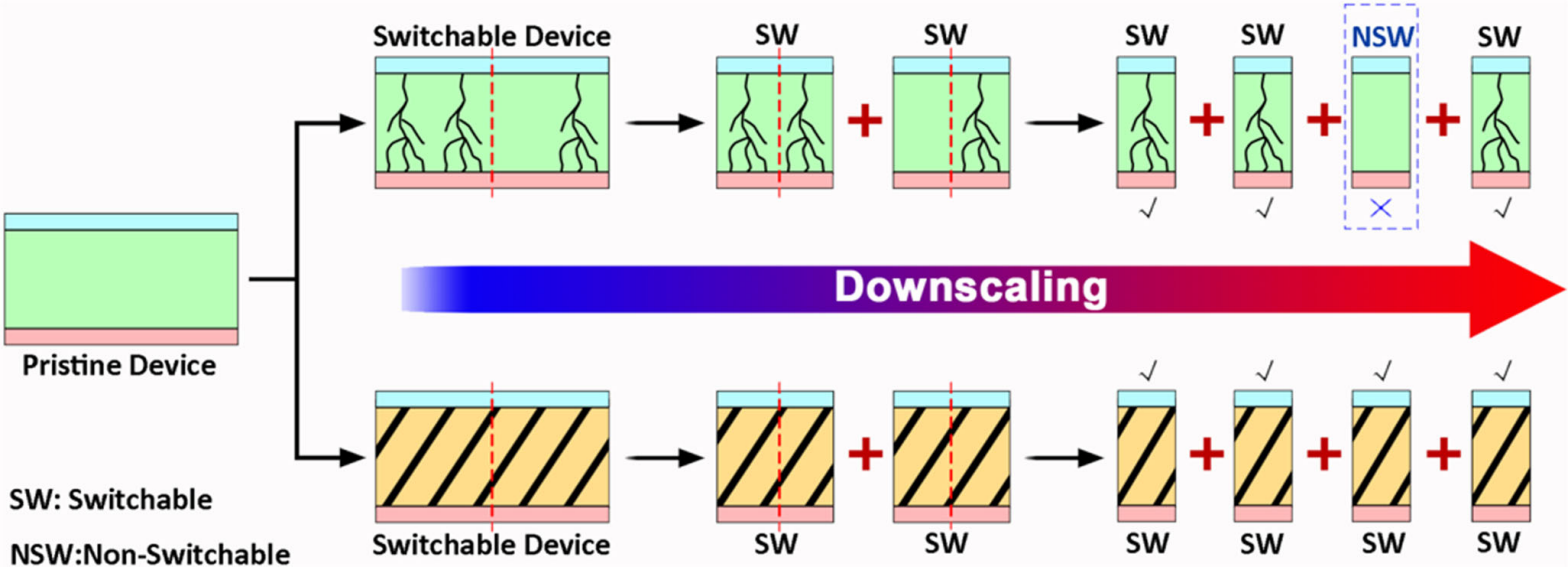
Localized and bulk switching mechanisms. Schematic illustration of the filamentary (upper panel) and bulk phase (lower panel) resistive switching phenomena in polymer memristor devices and their downscaling processes.

The first-time observation of resistive switching in polymer materials can date back to the 1970s^29^, wherein thermochemical pyrolysis of organic species led to the formation of carbon-based conductive filaments in glow-discharge polymerized thin films. Since electric conductivity of a material is proportional to the product of charge carriers’ concentration and mobility, changes in the carrier mobility along the carbon filaments can switch the device from a high resistance (OFF) state to a low resistance (ON) state. The pursuit of high-performance data storage technique in the new century reactivates the research interest in this area^30^, and various switching mechanisms of charge transfer, electrochemical redox reaction, conformational reconfiguration, ion migration, and etc have been proposed and employed for the design of polymer memory materials and devices. Regardless of the existence of different resistive switching mechanisms that either modulate the charge carriers’ concentration or mobility, or both, due to the structural anisotropy and inhomogeneity of most polymer materials, electric field usually distributes unevenly in the as-fabricated thin films. The molecular level defects associated with the polymer chains ends and folds, stacking faults, dislocations, as well as the macroscopic grain boundaries, voids and cracks, will both introduce unfavored charge trapping sites or scattering centers into the organic layers and converge the internal electric field around these regions^31^, giving rise to randomly and highly localized resistive switching characteristics (upper panel of Fig. 1). Downscaled fabrication of memristor cells onto samples with non-uniform microstructural features may result in devices with dissimilar electrical performance, leading to low production yield and reliability that are not suitable for practical applications. In case that the morphology of polymer thin films can be effectively controlled with certain degrees of ordering in its molecular packing and crystallinity, the distribution of internal electric field, molecular scale electronic procedures (e.g. generation and transport of free charge carriers), and thus the occurrence of resistive switching will be more homogeneous (lower panel of Fig. 1). The microstructural and electrical behaviors of such switching media do not vary with device dimensions and locations, which in turn will greatly enhance the stability, reliability, and production yield of polymer memristors during downscaling. The production of nanoscale devices will also lower the working current and energy consumption proportionally, therefore allowing the low-power operation for portable and edge computing applications.

**Fig. 2:**
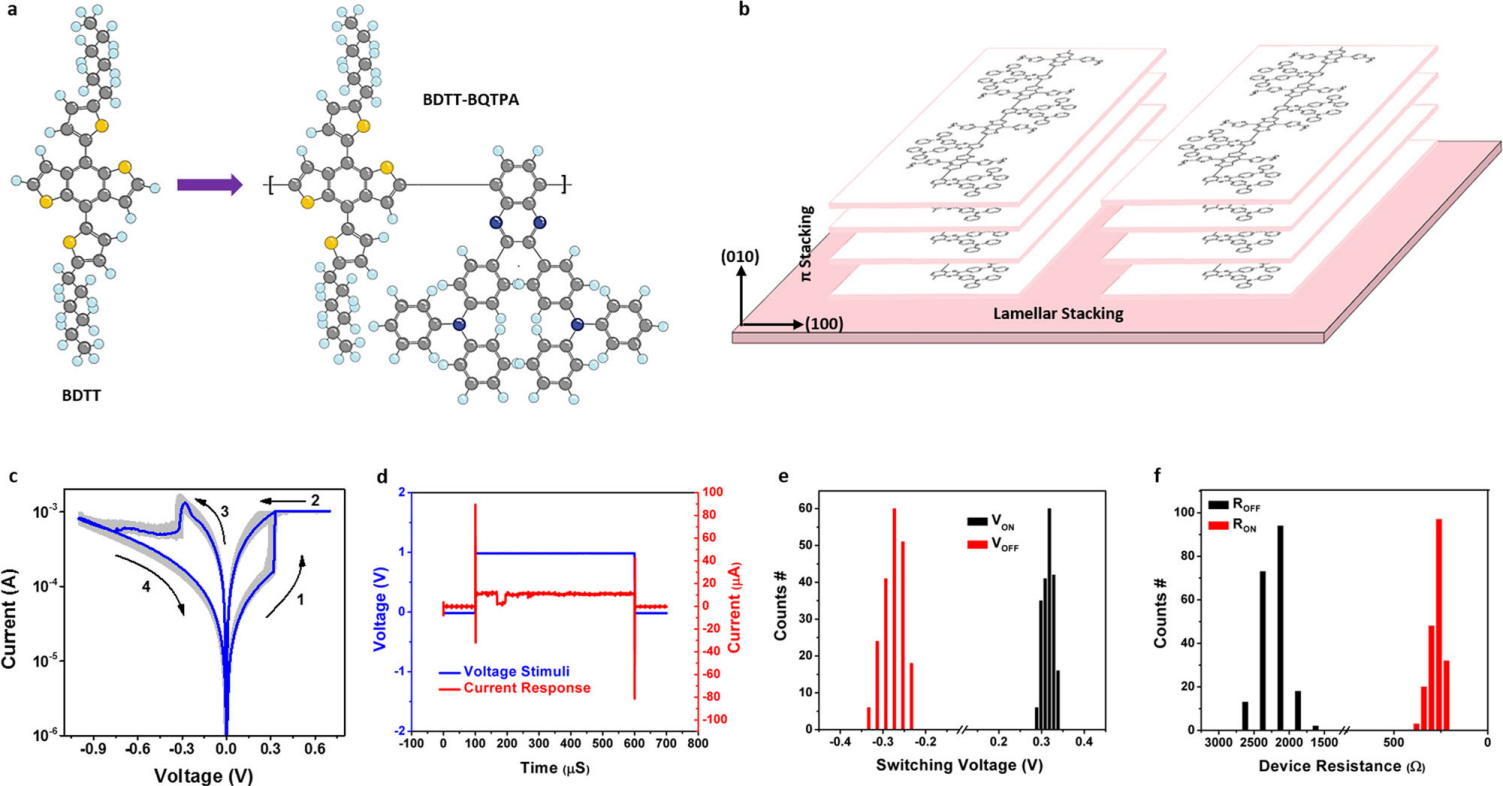
Molecular structure, stacking order, and uniform switching characteristics of the 2D conjugated polymer. **a** Molecular structures of the 2D conjugated BDTT electron donor and BDTT-BQTPA repeating unit. **b** Schematic illustration of the (010) direction *π*–*π* stacking and (100) direction lamellar stacking of the PBDTT-BQTPA 2D polymer chains. **c** Current–voltage curves collected from dozens of the Au/PBDTT-BQTPA/ITO devices. **d** Immediate device current of the PBDTT-BQTPA memristor in response to applied voltage with the amplitude of 1 V and pulse width of 500 μS. **e** Switching voltages and **f** device resistances distribution of the PBDTT-BQTPA memristor.

Efficient device performance directly depends on a series of electronic processes that are closely influenced by microstructural factors ranging from the molecular chemistry and short range ordering to device-scale alignments. Due to the highly asymmetric building blocks and the weak intermolecular van der Waals forces that hold the organic solids together, while introducing structural variations in the local molecular packing and larger scale order, it is difficult to receive stable and defect-free microstructures and morphologies. Nevertheless, structural assemblies must be controlled at a broad range of length scales from Ångstroms to centimeters simultaneously to impart optimal electronic characteristics. The past decade has witnessed a rapid progress in polymer optoelectronic devices, wherein detailed structure-property relationship has been established focusing on the influence of molecular arrangements and nano- to macro-scale structure on the optical and electrical events in organic photovoltaics (OPV) and light emitting diodes (OLED). Generally, high-performance solid-state optoelectronic materials usually have tendency to (at least partially) arrange in a periodic manner to promote charge carrier generation, transport, and collection procedures^25^. Herein, we adopted the two-dimensional *π*-conjugation strategy that can enhance the molecular planarity, packing ordering, and crystallinity of the polymer thin film to homogenize the distribution of the internal electric field and resistive switching in semicrystalline organic semiconductors^26–28^. A 2D-conjugated redox active polymer, PBDTT-BQTPA, was synthesized by Stille reaction as shown in Supplementary Fig. 1. Due to the considerable backbone conjugated length and the intra- and/or inter-chain charge transfer interaction between the adjacent D-A pairs (Fig. 2a), the main chain consisting of BDT electron donors and quinoxaline acceptors grants the polymer with high carrier mobility and semiconductor nature. In the meanwhile, the triphenylamine (TPA) groups tethered to the quinoxaline acceptor will be responsible for solid-state redox reaction and resistive switching under the stimuli of electric fields^12,32^. The occurrence of electrochemical reaction will result in the formation of polaron, solaron and/or other impurity energy levels inside the band gap of the initially semiconducting polymer material. This would make the charge carrier excitation from the highest occupied molecular orbital (HOMO) into the newly generated inter-gap energy levels easier, increasing the effective charge carrier concentration, and therefore leads to much improved conductivity of the thin film device. Most specially, the introduction of the alkylthienyl pendant substituents onto the 4 and 8 positions of the BDT chromophores may extend the intramolecular conjugation of the donor moiety from the original one-dimension along the polymer backbone into two-dimensional directions through the orthotropic conjugated side chains, which will in turn promote tight *π*–*π* stacking of the D-A pairs^33^. In ideal cases, the PBDTT-BQTPA thin film would demonstrate out-of-plane face-on molecular packing configuration and in-plane lamellar stacking of the polymer planes for a more homogeneous morphological feature that can homogenize the internal electric field for favored electronic transition scenarios (Fig. 2b).

The successful synthesis of 2D conjugated polymer PBDTT-BQTPA with an average molecular weight of 1.29 × 10^4^ and a polydispersity index of 1.46 was confirmed by the spectroscopic analysis (Supplementary Fig. 2 and Supplementary Note 1). The relatively narrow distribution of the polymer’s molecular weight, as well as its good solubility in organic solvents of toluene, chloroform (CHCl_3_), and dimethyl formamide (DMF), favors the preparation of high quality thin films for device applications. Being consistent with the n–π* transition of the hetero-atom containing thiophene and quinoxaline units, the PBDTT-BQTPA toluene solution shows UV-Visible absorption maximum at the wavelength of 332 nm (Supplementary Fig. 3 and Supplementary Note 2). An accompanying pair of the absorption shoulder peaks, associated with the *π*–π* transition of the conjugated polymer backbone, are also recorded at 310 nm and 370 nm, respectively^34^. Interestingly, in direct contrast with the much narrower absorption peak centered at 430 nm of the control sample PPH-BQTPA without 2D donor moiety, the moderate yet broad resonance band in the wavelength range of 460–630 nm nevertheless evidences the involvement of the planar BDTT chromophores and the effective extension of *π*-conjugation in the two-dimensional molecular system. Steady-state fluorescence spectra of PBDTT-BQTPA show prominent emission band at 612 nm in toluene, which moves to longer wavelength of 623 nm in chloroform and 648 nm in DMF, accordingly (Supplementary Fig. 4). The intensity of the emission band also decreases notably, along with the obvious broadening of the peak width. These phenomena are associated with the ground state intramolecular electron transfer between the BDTT and quinoxaline D-A pairs that endow the polymer with semiconductive nature^35,36^. In good agreement with the 2D conjugation strategy utilized to prepare PBDTT-BQTPA, its photoluminescence characteristics shows obvious red-shift when in comparison with that of the control sample PPH-BQTPA. PBDTT-BQTPA also exhibits good thermal stability, with a high onset decomposition temperature of 460 °C and maximum weight-loss temperature of 513 °C, for promising device applications (Supplementary Fig. 5).

### Switching-structure relationship of 2D conjugated polymer

Cyclic voltammetry reveals that both the 2D conjugated polymer PBDTT-BQTPA and the control sample PPH-BQTPA shows reversible redox activities. As plotted in Supplementary Fig. 6, the polymers show similar onset oxidation/reduction potentials of 1.13 V/−0.76 V and 1.17 V/−0.84 V, respectively, suggesting that the triphenylamine pendant unities rather than the thiophene-quinoxaline D-A pair makes major contribution to the electrochemical feature and potential memristive switching characteristic of the device. PBDTT-BQTPA carries a HOMO energy level of −5.54 eV and a lowest unoccupied molecular orbital (LUMO) level of −3.65 eV, respectively (Supplementary Note 3). Being consistent with the UV-Visible absorption spectra with an onset absorption edge of 624.5 nm and optical band gap of −1.99 V, the 2D conjugated polymer shows an energy band gap of 1.89 eV, which indicates its semiconductive nature suitable for electronic device applications. The influence of redox activities on the electronic characteristics of PBDTT-BTAPQ is also estimated by molecular simulations (Supplementary Fig. 7 and Supplementary Note 4).

In accordance with the electrochemical redox characteristics of PBDTT-BQTPA in forms of solid thin films, the Au/polymer/ITO device demonstrates bistable memristive switching behavior in both the d.c. current–voltage (*I*–*V*) sweeping and pulse mode measurements. In the present work, all the voltage stimuli were applied onto the Au electrodes while the ITO-coated conductive substrates were always grounded. Figure 2c illustrates the *I*–*V* curves collected from dozens of the as-fabricated devices. Referring to the redox behavior and energy band diagram of the sandwich structured device, hole injection from Au into the polymer layer will be an energetically favored process. When being scanned positively under low voltages, charge carriers do not gain sufficient energy to overcome the Schottky barrier at the Au/PBDTT-BQTPA interface, and the small device current is probably due to the tunneling. When the sweeping voltage exceeds the Schottky barrier height (0.44 eV), holes can be injected into the polymer layer and migrate towards the ITO cathode efficiently. As the voltage reaches 0.30 V, the TPA moieties of the macromolecules near the Au/polymer interface get electrochemically oxidized preferentially by the positively biased voltage, showing an abrupt current jump from the initial OFF (high resistance state, or HRS) state level of 1.6 × 10^−4^ A to the compliance preset of 10^−3^ A and turning the device to the ON (low resistance state, LRS) state. Neither the removal of the power supply nor reversing the scanning direction from 0.7 V back to 0 V can variate the energy band diagram of the oxidized polymer, leaving the transport properties of the polymer memristor unchanged in a non-volatile manner. Since the electrochemical oxidization of the TPA moieties propagates from the Au/polymer interface to the polymer/ITO interface, an asymmetric concentration gradient of the oxidized TPA^+^ species will be naturally established across the polymer layer and accounts for the bistable switching characteristics of the device. When the applied voltage with reversed polarity reaches −0.29 V, the electrons injected from the Au electrode begin to reduce the positively charged TPA^+^ species, extending from the upper Au/polymer interfacial area deep into the bottom polymer/ITO interface. Therefore, reprogramming of the TPA-containing polymer to the initial redox state and elimination of the impurity levels switch the device OFF, completing the writing-erasing operation of a polymer memristor. According to the formula *R* *=* *ρL*/*S*, where *R* is the resistance, *ρ* is the resistivity, *L* is the length, and *S* is active area of a resistor device, varying the polymer thin film thickness may result in different initial HRS resistance *R*_off_ of the memristor. Being consistent with this hypothesis, the devices with 80-nm-, 100-nm- and 120-nm-thick PBDTT-BQTPA thin film show similar and repeatable resistive switching characteristics, while the OFF state current decreases as the polymer layer thickness increases (Supplementary Fig. 8). On the other hand, the set voltage increases with the polymer film thickness, suggesting that the PBDTT-BQTPA memristor is driven by the electric field rather than the voltages.

The PBDTT-BQTPA memristor can be switched ON and OFF in both fast speed and reliable manner. When voltage stimulus with the amplitude of 1 V exceeding the switching ON voltage and pulse width of 500 µs has been applied onto the gold electrode, almost an immediate response in the device current can be observed (Fig. 2d). Further evaluation with pulse-measuring unit indicates that a responding time of no more than 32 ns (Supplementary Fig. 9), which is comparable to that of the state-of-the-art SRAM device and sufficient for data storage applications, can be recorded in the present PBDTT-BQTPA based memristor. It is noteworthy that assessment of the device responding time is practically limited by the sensitivity of the measuring equipments, and the actual switching speed of the 2D conjugated polymer may be even faster and suitable for in-memory computing applications. Being different from those devices that involves the rearrangement of the relative larger and heavier atomic and ionic species and thus show slow responding speed in µs to ms range^37–39^, the fast switching speed of the PBDTT-BQTPA device can be ascribed to the involvement of only electronic processes, e.g. electrochemical redox reaction of the TPA moieties. On the other hand, among dozens of the PBDTT-BQTPA devices being subjected to electrical tests, nearly 90% of them demonstrate repeated write-read-erase-read-rewrite capability shown in the cyclic switching characteristics of Fig. 2c, with uniform distribution of the programming voltages and ON/OFF state resistances (Fig. 2e, f). The device-to-device variations of the turn on/off voltages and ON/OFF resistances are 4.17%, 3.16%, 8.29%, and 6.25%, respectively. The record high production yield of the 2D conjugated polymer based memristors, together with the minor fluctuations of the switching parameters, confirms the effectiveness of the two-dimensional conjugation strategy for device performance optimization. Nevertheless, the symmetric switching behaviors of the PBDTT-BQTPA device, in terms of programming voltages with similar amplitudes, are beneficial for lowering the operational and architectural complexity of the memristor based integrated circuits. No obvious degradation of the ON or OFF state currents was observed after a testing period of 10^4^ s, or during continuous operations over 10^8^ cycles (Supplementary Fig. 10), revealing the excellent retention and endurance capability of the polymer memristor for practical applications. In good agreement with the thermal stability of the polymer material, the PBDTT-BATPA device shows repeatable switching characteristics at elevated temperatures of 50 °C, 75 °C, 100 °C, and 125 °C, exhibiting both promising retention and endurance behaviors (Supplementary Fig. 11).

**Fig. 3:**
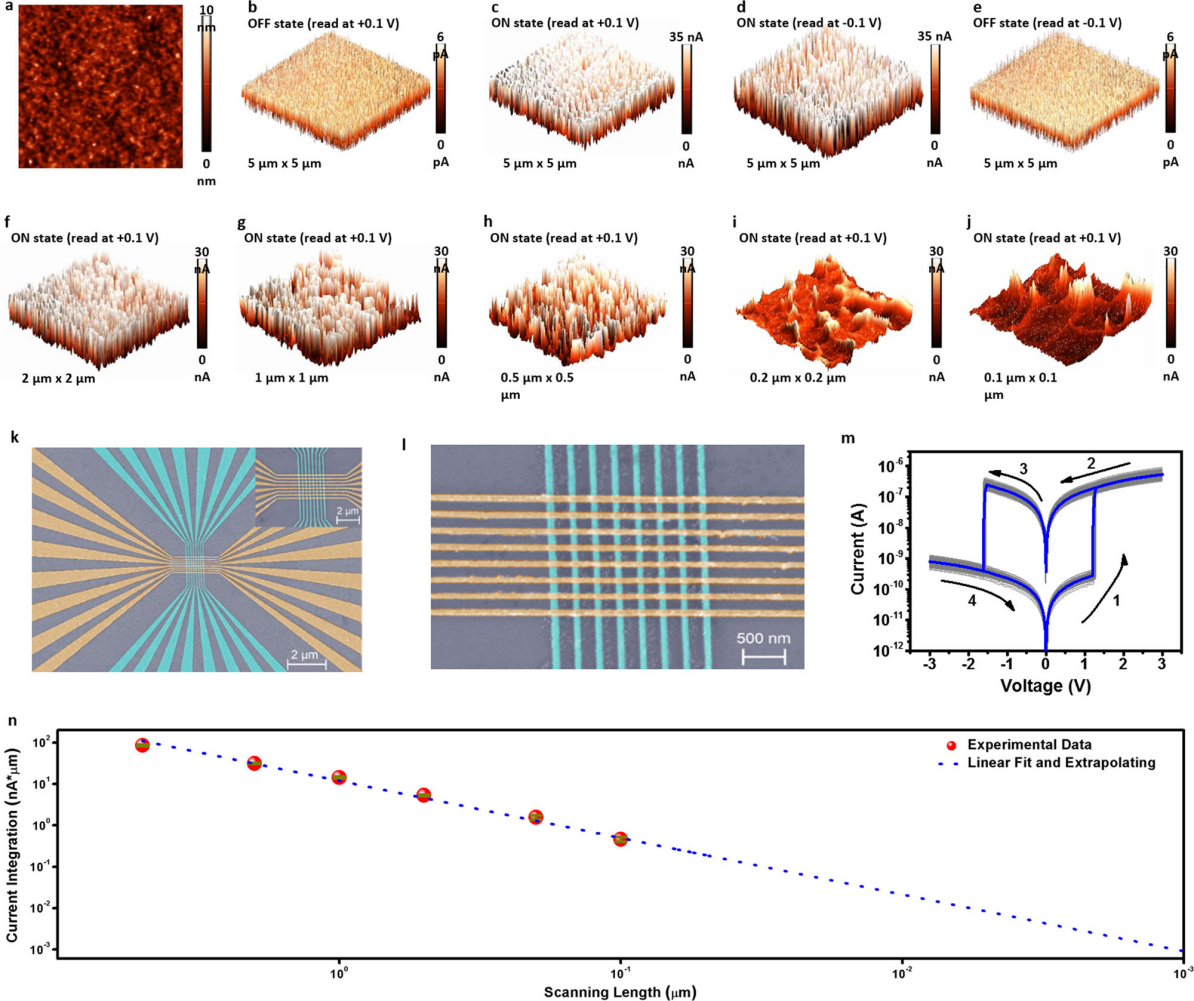
Bulk switching characteristics, scaling potential, and nano device performance of the 2D conjugated polymer. **a** Morphology and **b**–**e** ON/OFF state current mapping images of the PBDTT-BQTPA thin film over the scanning area of 5 μm × 5 μm. **f**–**j** ON state current mapping images over the scanning areas ranging from 2 μm × 2 μm to 0.1 μm × 0.1 μm. **k**, **l** Scanning electron images and **m** cyclic current–voltage characteristics of a nanometer scale PBDTT-BQTPA memristor device fabricated in an 8 × 8 crossbar array. Inset of **k** shows an entire view of the 8 × 8 memristor crossbar array. The electrode stripes are colored purposely for a better illustration. **n** Current integration vs. scanning length of the PBDTT-BQTPA thin film.

The resistive switching-microstructure relationship of the 2D conjugated polymer, and its scalability into nanometer scales, were evaluated through scanning probe microscopic analysis, wherein the morphology and local conducting/switching behavior were monitored in situ with a conductive-atomic force microscope (C-AFM) module. As displayed in Fig. 3a and Supplementary Fig. 12a, the PBDTT-BQTPA thin film spin-coated from toluene solutions onto the ITO substrate shows an atomic level smooth and featureless morphology, with an raw-mean-square (RMS) roughness of ~0.67 nm comparable to the molecular dimension of the BDTT-BQTPA repeating unit (~0.96 nm). The incorporation of the 2D donor BDTT with extended *π*-conjugation in both the polymer backbone and orthotropic sidechain directions is expected to enhance the coplanarity and intermolecular stacking of the thiophene-quinoxaline D-A pairs, therefore accounting for the as-observed structural uniformity and good device performance through better crystallinity of the organic thin films. Without the effective 2D conjugation and ordered stacking of the macromolecular chains, spin-coating of the PPH-BQTPA toluene solution exhibits featured surface morphology and worm like random structures appearing on top of the substrate (Supplementary Fig. 12b, c), which significantly deteriorates the stability of molecular level electronic procedure and reliability of the Au/PPH-BQTPA/ITO device. Although bipolar switching behaviors can also be observed in PPH-BQTPA devices occasionally (Supplementary Fig. 13a), in obvious contrast to that of the PBDTT-BQTPA samples, the *I*–*V* characteristic shows strong stochastic nature (Supplementary Fig. 13b) while the production yield is nevertheless no more than 40%. Figure 3b–e show the C-AFM current mapping images of the PBDTT-BQTPA thin film over a 5 µm × 5 µm area, before and after being subjected to the application of constant voltage stressing with the amplitude of ±1 V. As expected, the pristine PBDTT-BQTPA thin film shows good insulating feature with small leaking current not exceeding 2.5 pA. Switching to the ON state spatially homogeneously increases the conducting capability of the polymer layer, and the entire image shows remarkable and uniformly distributed current of more than 15 nA. In accordance with the bipolar and non-volatile resistive switching of the macroscopic Au/PBDTT-BQTPA/ITO device, removing the positive stressing and reading voltages, and reversing the polarity of the reading voltages do not influence the bulk phase ON state conducting characteristics apparently. Being different from the localized switching and filamentary conduction phenomena observed in most of the transition metal oxide and other organic based memristor devices, the universal resistive switching characteristics demonstrated in this work propagate through the entire 2D conjugated polymer layer and do not vary with sampling locations, which in turn greatly enhances the reliability and production yield of the memristors.

To extract the scaling extreme of the 2D conjugated polymer based memristor devices, we further investigated the local switching behavior with continuously shrunk sample dimensions during C-AFM observation. As depicted in Fig. 3f–j, stressing the PBDTT-BQTPA samples with different scanning areas all gives rise to the formation of conducting regions. Zooming in from the 2 µm × 2 µm sample to 0.1 µm × 0.1 µm sample reveals that the conducting behavior of the PBDTT-BQTPA thin film is homogeneous on sub-micrometer scales, with the distance between the neighboring switching spots to be ~40 nm. This indicates that the scaling limitation of the present polymer memristor can be extended reliably into 100 nanometer range with theoretical power consumption of ~10^−19^ J/bit. Such small size devices were fabricated in 8 × 8 crossbar arrays through electron-beam lithography technique and a lift-off approach, with the word and bit line widths and separations of 100 nm and 200 nm, respectively (Fig. 3k, l). Figure 3m shows 200 consecutive switching curves of a Pt/Ti/polymer/Au/Ti structured nano device in the 8 × 8 crossbar arrays, demonstrating good repeatability of the bistable characteristics. The circle-to-circle variations of the turn on/off voltages are 1.55% and 1.25%, while the ON/OFF resistances are (6.90 ± 1.46) × 10^6^ Ω and (4.60 ± 0.97) × 10^9^ Ω, respectively (Supplementary Fig. 14). Titanium was used to enhance the adhesion of the gold layer onto the SiO_2_/Si substrate and polymer layer. The different choice of electrode materials and thus contacts may only affect the injection barrier of the charge carriers from the electrode into the polymer layer, and thus slight change in the switching voltages. It is noteworthy that the nanoscale devices exhibit ON/OFF ratio (~10^3^) obviously lager than that of the micrometer scale devices. This can be understood in terms of the more uniform microstructure of the polymer thin film in smaller device area, as compared with the larger size organic layer with the minor presence of randomly orientated crystallites (see below for more details). Suppressing the structural inhomogeneity can avoid the introduction of defects or grain boundaries related leaking pathways, which in turn gives rise to the much smaller OFF state current and thus a larger ON/OFF ratio. Eliminating the converging center of the internal electric field also results in larger programming voltages (~1.25 V/−1.57 V) to turn the device ON and OFF. The actual power consumption of the device is ~5 × 10^−15^ J/bit.

To investigate the production yield of the nanometer scale memristors, we measured all the 64 devices in an 8 × 8 crossbar array. The *I*–*V* characteristics of the 64 memristor devices are provided in Supplementary Fig. 15, with 58 of them showing bistable switching capabilities. Based on this, the production yield for the nanoscale devices is estimated to be 90.6%, similar to that of the micrometer scale devices. The device-to-device variations of the turn on/off voltages are 5.57% and 4.57%, while the ON/OFF resistances are (5.76 ± 1.39) × 10^6^ Ω and (4.06 ± 1.18) × 10^9^ Ω (Supplementary Fig. 16). Again these results confirm the advantage of two-dimensional conjugation strategy for enhancing the device uniformity and reliability of polymer memristors. Upon integrating the sampling current along the scanning length of the C-AFM measurement, an obvious linear relationship between the current integration and device dimension can be plotted as shown in Fig. 3n. Proportional extrapolating suggests ultra-small working current, which is suitable for portable and low-power applications, can be achieved in sub-10-nm organic memristors.

**Fig. 4:**
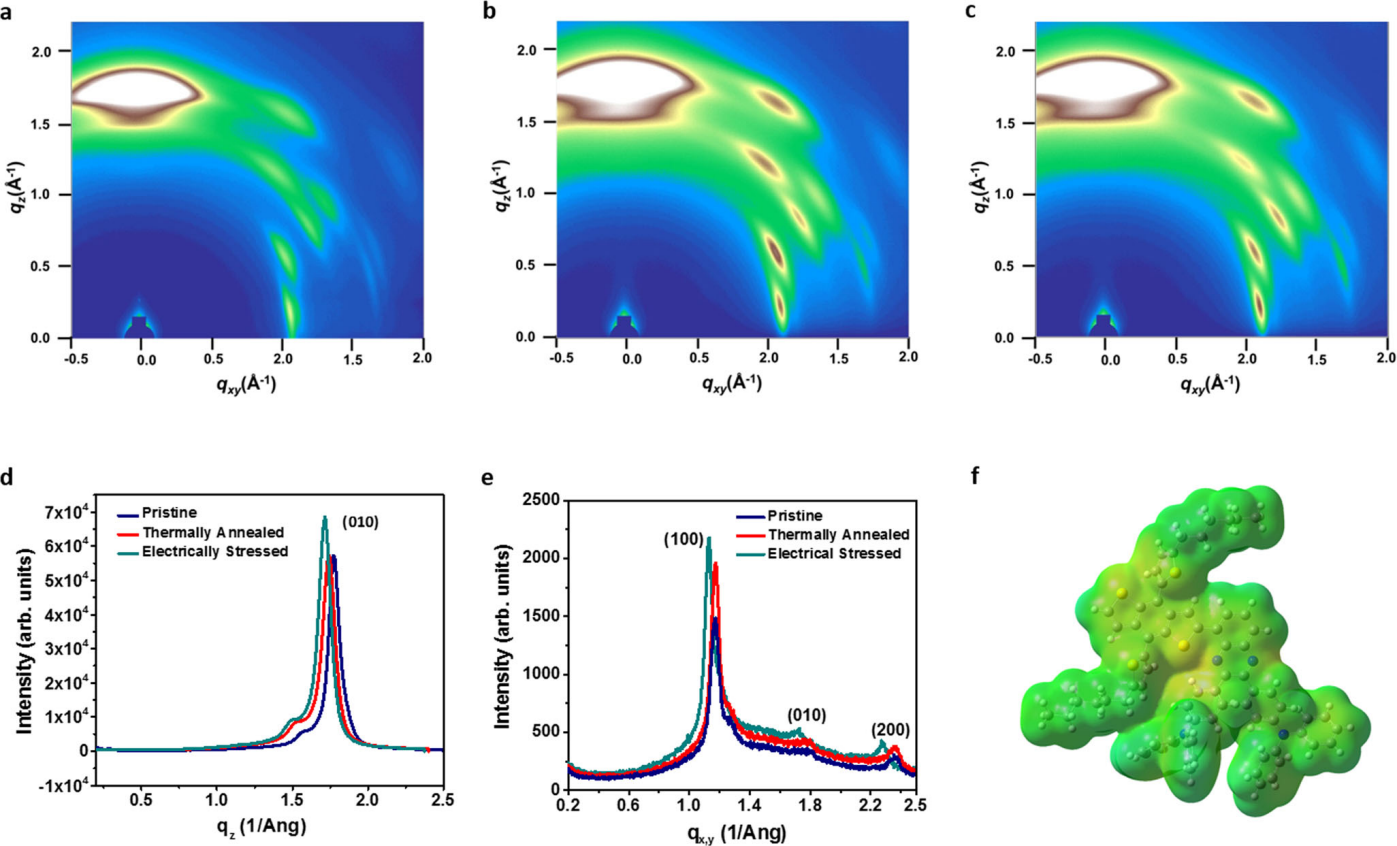
Crystallinity of the 2D conjugated polymer. GIWAXS images of the PBDTT-BQTPA thin film **a** in its pristine state, **b** under thermal annealing and **c** upon being subjected to voltage stressing. Line cuts of the GIWAXS of the PBDTT-BQTPA thin film in **d** the out-of-plane direction and **e** the in-plane direction. **f** Electrostatic potential (ESP) surface of the repeating unit of PBDTT-BQPTA derived from molecular simulation.

The correlation between the structural uniformity, occurrence of the homogeneous resistive switching phenomena, and the 2D conjugation strategy of PBDTT-BQTPA polymer, are further confirmed through microstructure investigation with grazing incident wide-angle X-ray scattering (GIWAXS) measurements and molecular simulations^31^. Figire 4a–e display the GIWAXS images and plots of the PBDTT-BQTPA film in its pristine state, under thermal annealing at 120 °C for 10 min and after being subject to voltage stress with the amplitude of 5 V for 3 min, respectively. All the polymer thin films were prepared by spin-coating approach. During fabrication, the polymer solution is dropped onto the spinning substrate and the organic solvents evaporate during spinning. As time passes, the concentration of the polymer molecules increases and they begin to aggregate. Due to the intra-/intermolecular *π*–*π* interaction between the electron donor–acceptor pairs, the macromolecule chains aggregate in an ordered manner and crystallize gradually. Bright scattering pattern with strong and narrow (010) peak at the *q*_z_ of 1.77 Å^−1^, as shown in Fig. 4a, d, reveal the semicrystalline nature of the pristine polymer thin film and strong preference of the face-on orientation of the conjugated backbone in the out-of-plane direction. The *π*–*π* stacking distance of the PBDTT-BQTPA crystallite, ~3.54 Å, is similar to that of its BDTT analog J71 as reported in the literature^27^. Characteristics of the in-plane ordering with much weaker (100) peak intensity is also observed at the *q*_xy_ of 1.17 Å^−1^ (Fig. 4e), indicating the presence of lamellar edge-on configuration of the polymer planes as proposed in Fig. 1. The lamellar stacking distances of 5.36 Å, which is much smaller than the 17.44 Å of the J71 polymer, suggests that the PBDTT-BQTPA film has a lateral compacter packing mode of the polymer crystallites. Minor azimuthal distributions of the scattering patterns also exist in the GIWAXS pattern, which correspond to the random orientation of the crystallites in PBDTT-BATPA thin films and can be ascribed to the steric effect of the propeller shape triphenylamine redox pendants and alkylthienyl substituents. It is noteworthy that both the *π*–*π* and lamellar stacking of the PBDTT-BQTPA backbone can be enhanced through both thermal and electrical annealing (Fig. 4b, c), leading to better crystallinity yet negligible expansion of the polymer stacks. In comparison to the amorphous or highly polycrystalline (e.g., PPH-BQTPA) counterparts, the crystallite size of the PBDTT-BQTPA thin film with improved crystallinity and homogenized microstructure should be much larger, which in turn can suppress the formation of the molecular level defects of chain ends, folds, stacking faults, dislocations, and the grain boundaries, voids, and folds. Arising from the combination of the preferred face-on orientation and tight stacking of the polymer segments, made possible through 2D conjugation with the coplanar thiophene-quinoxaline D-A pair (Fig. 4f), converging of the electric field around these less amount grain boundaries may be effectively hindered, leading to the occurrence of bulk phase resistive switching across the entire sample with enhanced uniformity and greater production yield of the polymer memristor devices.

### In-memory logic, arithmetic, and neuromorphic computing

The non-volatile reconfiguration of memristors’ resistance provides physical mechanism for the execution of in-memory logic operations^40^. We have demonstrated in previous work that the redox activities and memristive switching characteristics of polymer devices can be used to activate the simple logic operators^12^, while the record-high production yield of 90% and promising reliability achieved in the present PBDTT-BQTPA memristors allow us to conduct the array scale implementation of all 16 Boolean logic and arithmetic functions. For instance, the NAND, NOR, AND, OR, and 10 other logic operations can be achieved in three sequential steps of initializing and writings within a single device (Supplementary Fig. 17 and Supplementary Note 5)^41^, while the incorporation of a pair of anti-serially connected memristors grants the implementation of the remaining XOR and XNOR logic operations^42,43^.

**Fig. 5:**
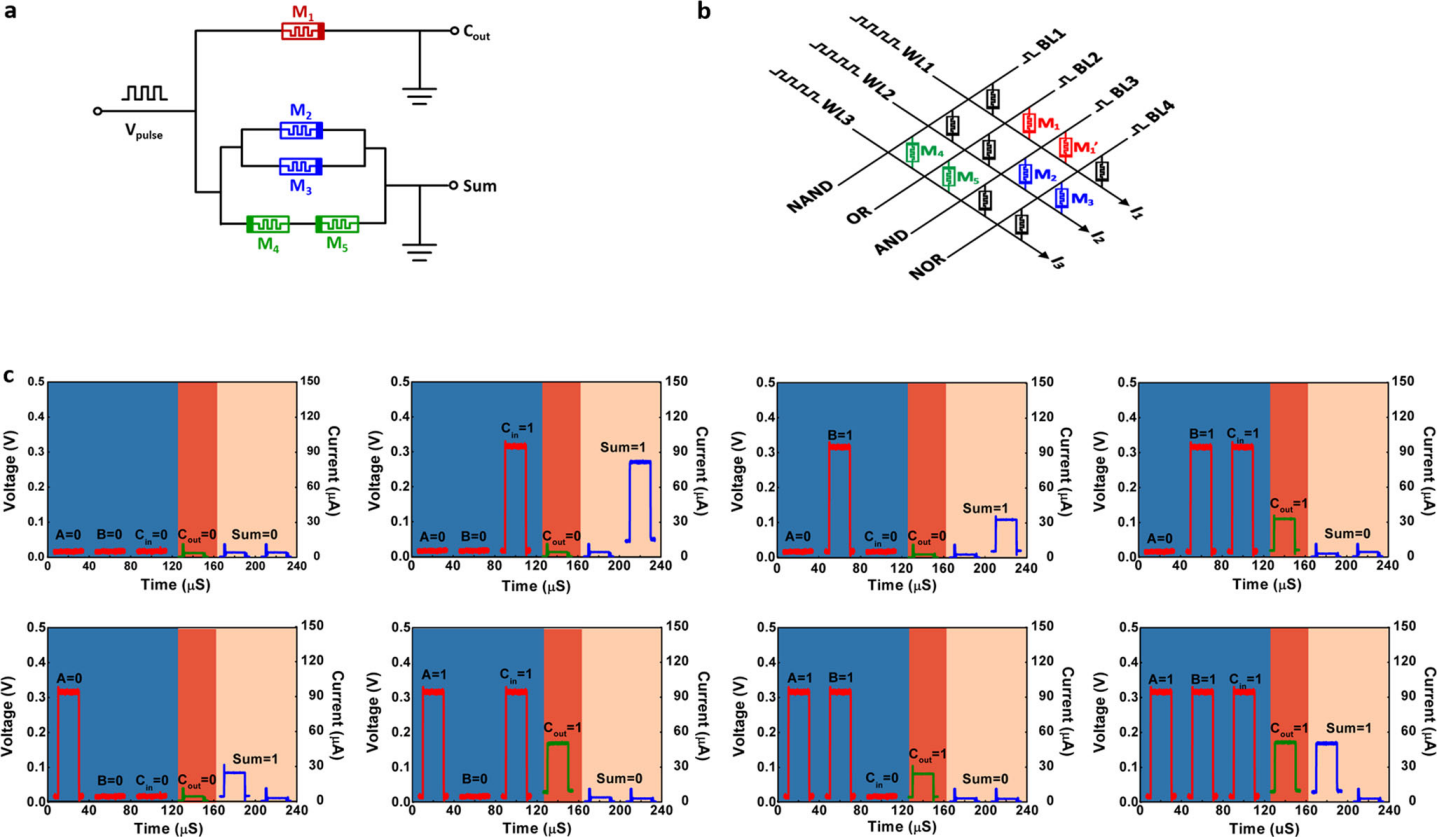
Logic operation based on the 2D polymer memristor devices. **a** Circuit design, **b** memristor array representation, and **c** experimental datasets of a parallel 1-bit full adder. The blue area of **c** represents the logic inputs, while the orange and yellow regions represent the bit-carry and sum outputs.

Functional demonstration of the arithmetic-logic module, which is the core unit of a computer CPU, is also made possible by the combination of the NAND, OR, AND, and NOR operators with a 4 (bit line, BL) × 3 (word line, WL) PBDTT-BQTPA memristor array^44,45^. Figure 5a, b show the design and memristor array representation of the parallel 1-bit full adder circuit involving only five polymer devices. Signal A is utilized to define the initial state of the memristors, while signals B and C perform the logic operations through the bit lines of the memristor array (Supplementary Table 1). Note that although the inherent hardware reconfigurability allows the execution of OR and AND operations through a single memristor without changing the circuit architecture, M1 and M1′ are still used in the array instead to represent the respective operators more clearly. In such memristor circuit, M1/M1′ is designed for the bit-carry function and M2 to M5 carry out the summing operation. Supplementary Table 2 lists operation methodology of the full adder, wherein the bit-carry (*C*_out_) and sum (*Sum*) outputs of the memristor array are represented by the WL1 current (*I*_1_) and sum of the WL2 and WL3 currents (*I*_2_ + *I*_3_), respectively. To implement the carry operation, M1 is initialized to LRS while *A* = 0 or 1 is inputted to reconfigure the device for AND or OR operator, accordingly. For the addition calculation, M2 to M5 devices are set to HRS, HRS, LRS, and LRS in the first stage. With the input of *A* = 0, the logic states of M2, M3, M4, and M5 remain unchanged. The parallel connection between M2 and M3 leads to the HRS (with low *I*_2_) output signals, while the serial connection between M4 and M5 results in the respective ON and NAND operations accordingly. When the input *A* is set to 1, the resistance states of the M2–M4 devices are reversed while M5 remains unchanged. The NOR and AND functions are performed by M2 and M3, respectively, while the WL3 shows low *I*_3_ of the M4 and M5 HRS states. The experimental outcomes of the summing operations, read by a small voltage of 0.01 V and plotted in Fig. 5c, coincide with the truth table and verify the successful implementation of a-bit full adder circuit with the present polymer memristor array.

**Fig. 6:**
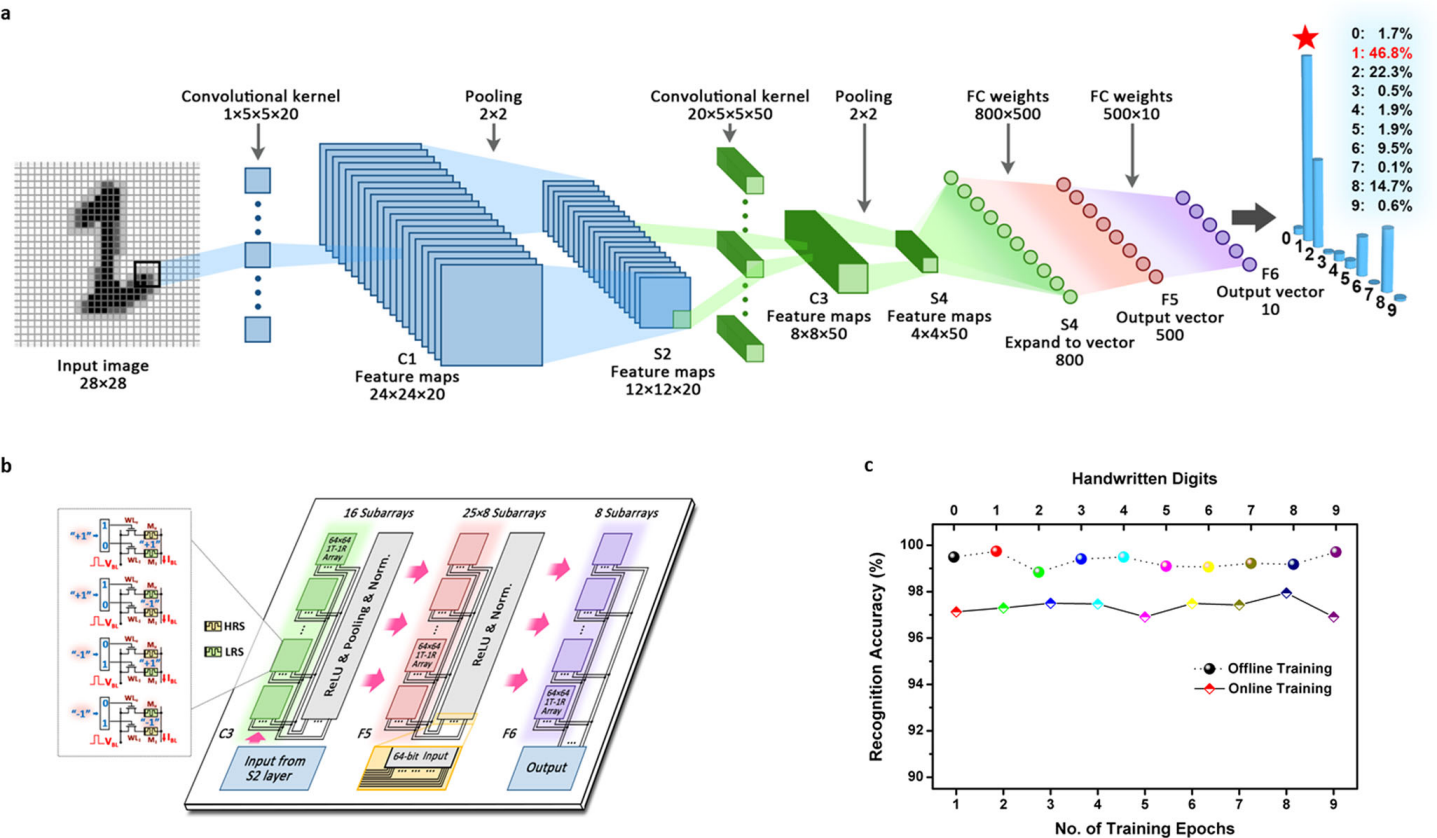
Simulated neuromorphic pattern recognition based on the 2D polymer memristor devices. **a** Architecture of LetNet-5 binary neural network for handwritten digits recognition. **b** Illustration of the PBDTT-BQTPA polymer memristor arrays in the binary neural network. Left panel shows the execution of XNOR operation with two bit-cells, wherein each bit-cell of the 64 × 64 subarrays is composed of one polymer device and one NMOS transistor. **c** Recognition accuracies of the handwritten numeral images from the MNIST dataset through offline and online trainings.

Beyond the in-memory Boolean logic and arithmetic operations for general purpose computing, the resistive switching characteristics of the present PBDTT-BQTPA devices can also be used to construct memristive array hardware accelerator in artificial neural network (ANN) for pattern recognition tasks. Although that deep learning techniques using conventional convolutional neural networks (CNNs) and recently well studied multilevel resistive switching memristors have demonstrated remarkable performance in artificial intelligence applications of computer vision and speech recognition^46–51^, their huge demands on memory capacity and computing power still make them less competitive for resource-limited edge computing in embedded systems or mobile devices. The non-ideal analog synaptic weight characteristics, e.g. deviation from the linear and precise weight updating in limited dynamic range, nevertheless, will introduce additional risks of computational accuracy degradations. In this work, we build a binary neural network (BNN) with the present two-state resistive switching polymer memristors, wherein the synaptic weights and neuron activations are both binarized to +1 or −1 instead^52,53^. As shown in Fig. 6a, the LeNet-5 model is employed to construct the binary neural network for handwritten digits recognition. The convolutional layer C3 and fully connected layers F4 and F5 are composed of the binary memristors, while the activation functions, pooling layers, and the normalization layers of the hidden layer remain unchanged in the Pytorch framework (Supplementary Table 3). Through mathematical modeling of the experimental *I*–*V* characteristics to extract the switching parameters of the memristor (Supplementary Fig. 18 and Supplementary Note 6), definition of the logic “0” and “1” in accordance to the HRS/LRS resistances, as well as the binary weights of +1 and −1, are assigned to the combination of two polymer devices and stored in multiple subarrays of 64 × 64 memristors (Fig. 6b and Supplementary Table 4). Being different from the conventional CNN networks, BNN uses bit-wise XNOR or XOR as the fundamental operator to replace the time-consuming multiplication-and-accumulation (MAC) operations of the convolutional and fully connected layers (Supplementary Note 7). Simulation of the offline supervised learning using the experimental memristive characteristics and 180 nm field-effect transistors from the Semiconductor Manufacturing International Corporation (SMIC) reveals that over 99.23% of the recognizing accuracy can be achieved for 10,000 (28 × 28 pixel) images of the handwritten numerals from the MNIST datasets (Fig. 6c)^54^. This result suggests that binary neural networks are capable of achieving satisfying performance on representative pattern recognition tasks, wherein the bit-wise operation (involving only binary weights of +1 and −1, as compared to the floating-/fixed-point precision) can lead to significantly reduced memory and computing resource usages. Online learning simulation also gives a high recognizing rate of 97.13% after 1 epoch training with 60,000 images.

## Discussions

To summarize, we demonstrate that by using the 2D conjugation strategy, 90% production yield of the organic memristors can be achieved with PBDTT-BQTPA redox active polymers. Through enhancing the crystalline uniformity of the polymer thin film via coplanar thiophene- quinoxaline D-A pair and ordered *π*–*π* stacking of the macromolecule backbone, homogeneous resistive switching occurs across the entire organic layer, leading to switching parameter variation down to 3.16–8.29%, record-high production yield approaching 90%, and potential scalability into 100 nm scale with tiny power consumption of ~10^−15^ J/bit. The high production yield and reliability achieved in the present PBDTT-BQTPA memristors allow us to conduct array scale in-memory computing operations, wherein all the 16 Boolean logic operations and 1 bit full adder circuit can be implemented physically. More importantly, we demonstrate that the two-state switching organic memristors can also be used to construct binary neural networks, which shows promising recognition accuracy of 99.23%, comparable to that of the mature deep learning techniques. Nevertheless, it is noteworthy that replacing the propeller-shaped TPA pendants with other planar redox active moieties may further improve the crystallinity of the polymer thin films, therefore promoting the downscaling of the organic devices to the technical extreme of the state-of-the-art lithographic production lines.

## Methods

### General characterization

^1^H nuclear magnetic resonance (NMR) spectra were recorded on a Bruker 400 spectrometer at 400 MHz in deuterated solution with tetramethylsilane (TMS) as reference for the chemical shifts. Molecular weights were determined with a Waters 2690 gel permeation chromatography (GPC) using a polystyrene standards eluting with tetrahydrofuran. Thermogravimetric analyses (TGA) were carried out using Pyris 1 TGA. The UV-Visible absorption spectral measurements were recorded on a Shimadzu UV-2450 spectrophotometer. Fluorescence spectra were measured on a HORIBA JOBIN YVON Fluoromax-4 spectrofluorometer. Cyclic voltammetry (CV) measurements were performed on a model CHI 650D electrochemical workstation with tetrabutyl ammonium hexafluorophosphate (0.10 M) in acetonitrile as the supporting electrolyte, a platinum disk as the working electrode, an Ag/AgCl electrode as the reference electrode, and a Pt wire as the counter electrode, respectively. Dry HPLC grade acetonitrile was degassed with high purity Argon to eliminate possible interference arising from the environmental moisture and oxygen species.

Grazing-incident wide-angle X-ray scattering (GIWAXS) measurements where performed at the PLS-II 9A USAXS beam line of the Pohang Accelerator Laboratory in Korea. X-rays coming from the in-vacuum undulator (IVU) were monochromated with the wavelength of 1.10994 Å using a double crystal monochromater and focused both horizontally and vertically using K-B type mirrors. The beam size is 450 (H) × 60 µm^2^ in fwhm at sample position. The GIWAXS sample stage was equipped with a 7-axis motorized stage for the fine alignment of sample, and the incidence angle of X-ray beams was set at ~0.13^o^ to 0.135^o^. GIWAXS patterns were recorded with a Rayonix SX 165 2D CCD detector with an irradiation time of 6–9 s. The diffraction angles were calibrated using a sucrose standard (monoclimic, P21, *a* = 10.8631 Å, *b* = 8.7044 Å, *c* = 7.7624 Å, and *ß* = 102.938^o^) with the sample-to-detector distance of ~231 nm. Atomic force microscopy (AFM) measurements were performed on a Solver P47-PRO (NT-MDT Co., Moscow, Russia) microscope to monitor the surface morphology of the polymer thin films.

### Molecular simulation

All density functional theory (DFT) and time-dependent DFT (TD-DFT) calculations were performed using the Gaussian 09 package^55,56^. For better comparison between the neutral and charged compounds, geometry optimization and excited-state calculation are performed in singlet and close shell. The B3LYP functional was used for geometry optimization in the ground state, with the 6–31 G(d) basis set for the C, H, N, and S atoms. All geometry optimization was done in the gas phase.

### Device fabrication and characterization

The electrical properties of the PBDTT-BQTPA and PPH-BQTPA were evaluated in devices with structure of Au/polymer/ITO. Commercially available indium tin oxide (ITO) coated glass substrates with the size of 1 cm × 1 cm were carefully cleaned with detergent, deionized water, ethanol, acetone, and 2-propanol in an ultrasonic bath, each for 15 min in that order. A 50 µL of PBDT-BQTPA or PPH-BQTPA solution (10 mg/mL) in toluene was spin-coated on the pre-cleaned ITO substrate at 600 rpm for 10 s and then at 2000 rpm for 40 s. Afterwards, the obtained devices were thoroughly dried at 80 °C in vacuum overnight. Thickness of the films was measured to be ~100 nm by a step-profiler. To complete fabricating the device, Au top electrodes (120 nm in thick, 0.4 × 0.4 mm^2^ in area) were thermally deposited onto the surface of the polymer layers through a shadow mask at 10^−7^ Torr. Nanometer scale devices were fabricated through the electron-beam lithography technique and a lift-off approach. Crossbar electrode strips, with the width of 100 nm and separation of 200 nm between each other, were formed on SiO_2_/Si substrate. The bottom electrode strips were patterned by electron-beam lithography with a Vistec EBPG-5200^+^ Electron-beam lithography system, and E-beam evaporation of a 20-nm Au layer on the top of 10 nm Ti adhesion layer on a Denton Electron Beam Evaporator. After lift-off, a 50 µL of PBDT-BQTPA solution (5 mg/mL) in toluene was spin-coated onto the Si wafer at 4000 rpm for 40 s, and then was thoroughly dried at 80 °C in vacuum overnight. Finally, the top electrodes consisting of 15 nm Ti and 45 nm Au were patterned and deposited using electron-beam lithography, e-beam evaporation and lift-off similarly. All electrical measurements of the as-fabricated devices in this work were performed on a Keithley 4200 semiconductor parameter analyzer equipped with a pulse-measuring unit in ambient condition with no further protection. In the C-AFM measurement, the PBDT-BQTPA film was spin-coated onto the ITO-coated glass substrate. Biased voltage was applied to the polymer film directly by using the Pt-coated C-AFM tip as a movable top electrode.

### Simulation of binary neural network

The LeNet-5 binary neural network mainly consists of an input layer, an output layer and a hidden layer containing sequentially the convolutional layer C1, pooling layer S2, convolutional layer C3, pooling layer S4, fully connected layers F5 and F6, respectively (as shown in Fig. 6a and Supplementary Table 3). In all, 28 × 28 pixel images of the handwritten digits from the MNIST datasets are used as input feature maps, which are subsequently convoluted in C1 layer with 20 convolutional kernels. The size of each kernal in C1 is 5 × 5 × 1, giving rise to 20 output feature maps (or input feature maps of the following pooling layer S2) with the size of 24 × 24 pixel. The size of the C1 output feature maps is determined as 28−5 + 1 = 24. The total numbers of the trainable parameters in this layer is 5 × 5 × 20 + 20. In the first pooling layer S2, max_pool subsampling with the pooling size of 2 × 2 has been performed, resulting in 20 filtered maps with the smaller size of 12 × 12. These 12 × 12 maps are then projected onto the binary layer C3 containing 50 convolutional kernels. Each of the C3 kernel is (5 × 5) × 20 in size and the output feature map has a furthered shrunk size of 8 × 8. In this convolutional layer, the number of the trainable parameters is (5 × 5) × 20 × 50 + 50. The second pooling layer S4 also performs the max_pool operation with a pooling size of 2 × 2. The size of resultant 50 output feature maps is 4 × 4. Finally, the fully connected layers F5 and F6 integrate the highly abstracted features of the convoluted input images and generate a probability number for each of the 10 handwritten digit possibilities. According to the as-obtained probability numbers, the output layer will recognize the exact classification of the input handwritten digit images.

The performance of the as-formed binarized LeNet-5 network is evaluated on the basis of Pytorch^57^, PyOPUS^58^, and Cadence spectre^59^ platforms. The convolutional layer C3 and fully connected layers F4 and F6 are mapped to memristive subarrays (Supplementary Note 6) and simulated in Cadence spectre, while the rest of the network is simulated using python (Pytorch framework). PyOPUS is used as interface between Pytorch and Cadence spectre to exchange feature maps. During simulation, PBDTT-BATPQ memristors with the device size of 400 × 400 nm^2^ have been used.

## Supplementary information

Supporing Information

## Data Availability

The authors declare that the main data supporting the findings of this study are available within the article and its Supplementary Information files. Extra data are available from the corresponding author upon request.
