# Supplementary material for: 90% yield production of polymer nano-memristor for in-memory computing
Source: Nat Commun. 2021 Mar 31;12:1984. doi: 10.1038/s41467-021-22243-8 (PMC8012610; doi:10.1038/s41467-021-22243-8)
Supplement: Supplementary file 1 — Supporing Information [file 41467_2021_22243_MOESM1_ESM.pdf]

Supporting information

90% Yield Production of Polymer Nano-Memristor for In-Memory Computing

Bin Zhang[1†], Weilin Chen[2†], Jianmin Zeng[3†],Fei Fan[1,2,4], Junwei Gu[5], Xinhui Chen[2], Lin Yan[3], Guangjun Xie[3], Shuzhi Liu[4], Qing Yan[1], Seung Jae Baik[6], Zhi-Guo Zhang[7], Weihua Chen[7], Jie Hou[1], Mohamed E. El-Khouly[8], Zhang Zhang[*3], Gang Liu[*2], and Yu Chen[*1]

[1]Key Laboratory for Advanced Materials and Joint International Research Laboratory of Precision Chemistry and Molecular Engineering, Feringa Nobel Prize Scientist Joint Research Center, School of Chemistry and Molecular Engineering, East China University of Science and Technology, Shanghai 200237, China.

[2]School of Electronic Information and Electrical Engineering, Shanghai Jiao Tong University, Shanghai 200240, China.

[3]School of Electronic Science and Applied Physics, Hefei University of Technology, Hefei 230601, China.

[4]School of Chemistry and Chemical Engineering, Shanghai Jiao Tong University, Shanghai 200240, China.

[5] Shaanxi Key Laboratory of Macromolecular Science and Technology, School of Chemistry and Chemical Engineering, Northwestern Polytechnical University, Xi' an, Shaanxi, 710072, PR China

[6]Department of Electrical, Electronic and Control Engineering, Hankyong National University, Anseong-si, Gyeonggi-do, 17578, Korea

[7]Green Catalysis Center and College of Chemistry, Zhengzhou University, Zhengzhou 450001, China.

[8]Institude of Basic and Applied Sciences, Egypt-Japan University of Science and Technology (E-JUST), Alexandria, Egypt

All correspondence and request for materials should be addressed to: chentangyu@yahoo.com (Prof. Yu Chen), gang.liu@sjtu.edu.cn (Prof. Gang Liu), zhangzhang@hfut.edu.cn (Prof. Zhang Zhang).

[†]These authors contributed equally to this work.

Supplementary Figures

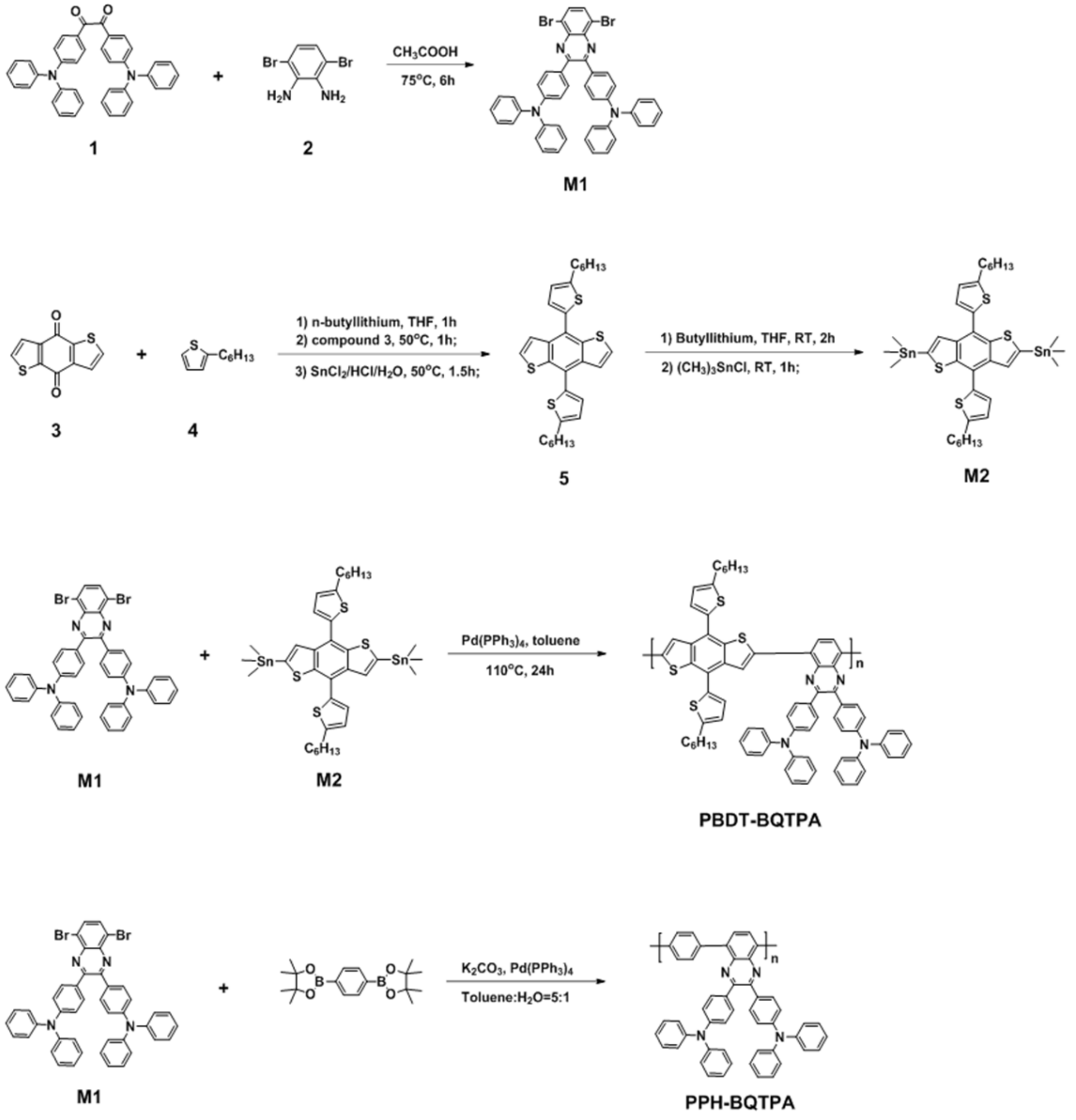

Supplementary Figure 1. Materials synthesis. Synthetic routes of PBDTT-BQTPA and PPH-BQTPA.

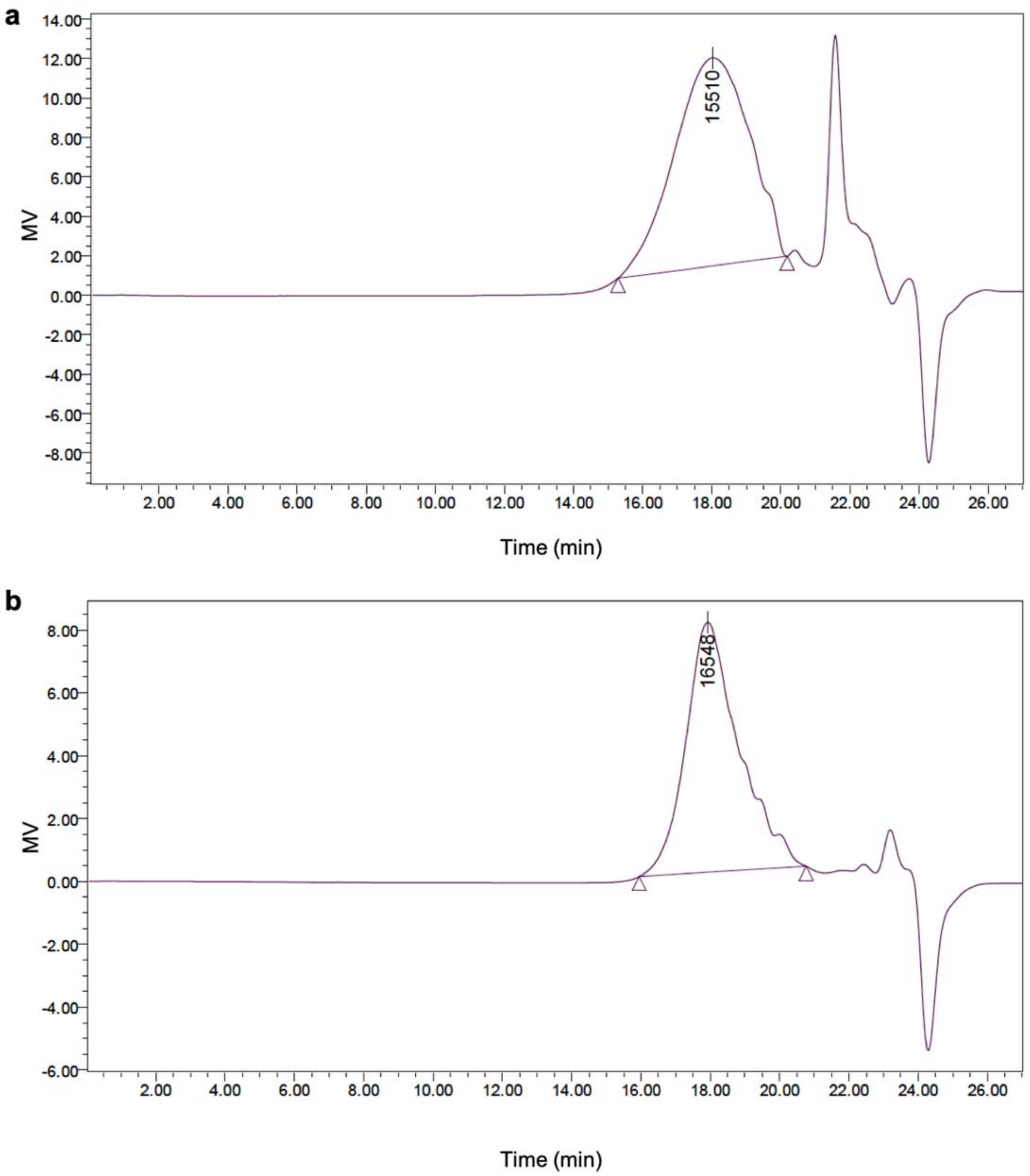

Supplementary Figure 2. Gel permeation chromatography spectra. (a) PBDTT-BQTPA and (b) PPH-BQTPA, respectively.

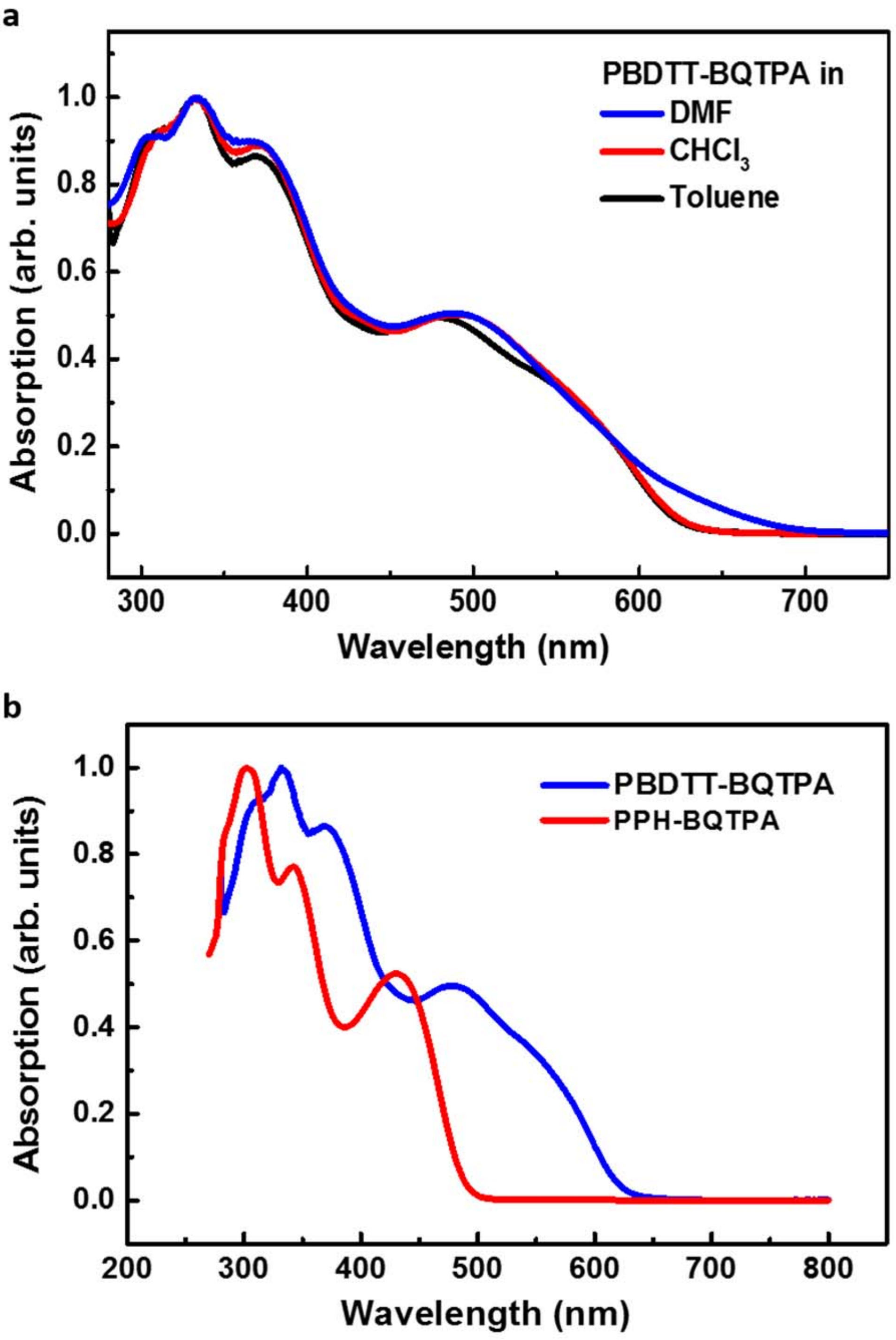

Supplementary Figure 3. UV-Visible spectra. (a) PBDTT-BQTPA in toluene, chloroform and dimethyl formamide (DMF) solutions and (b) PBDTT-BQTPA and PPH-BQTPA in toluene solutions, respectively.

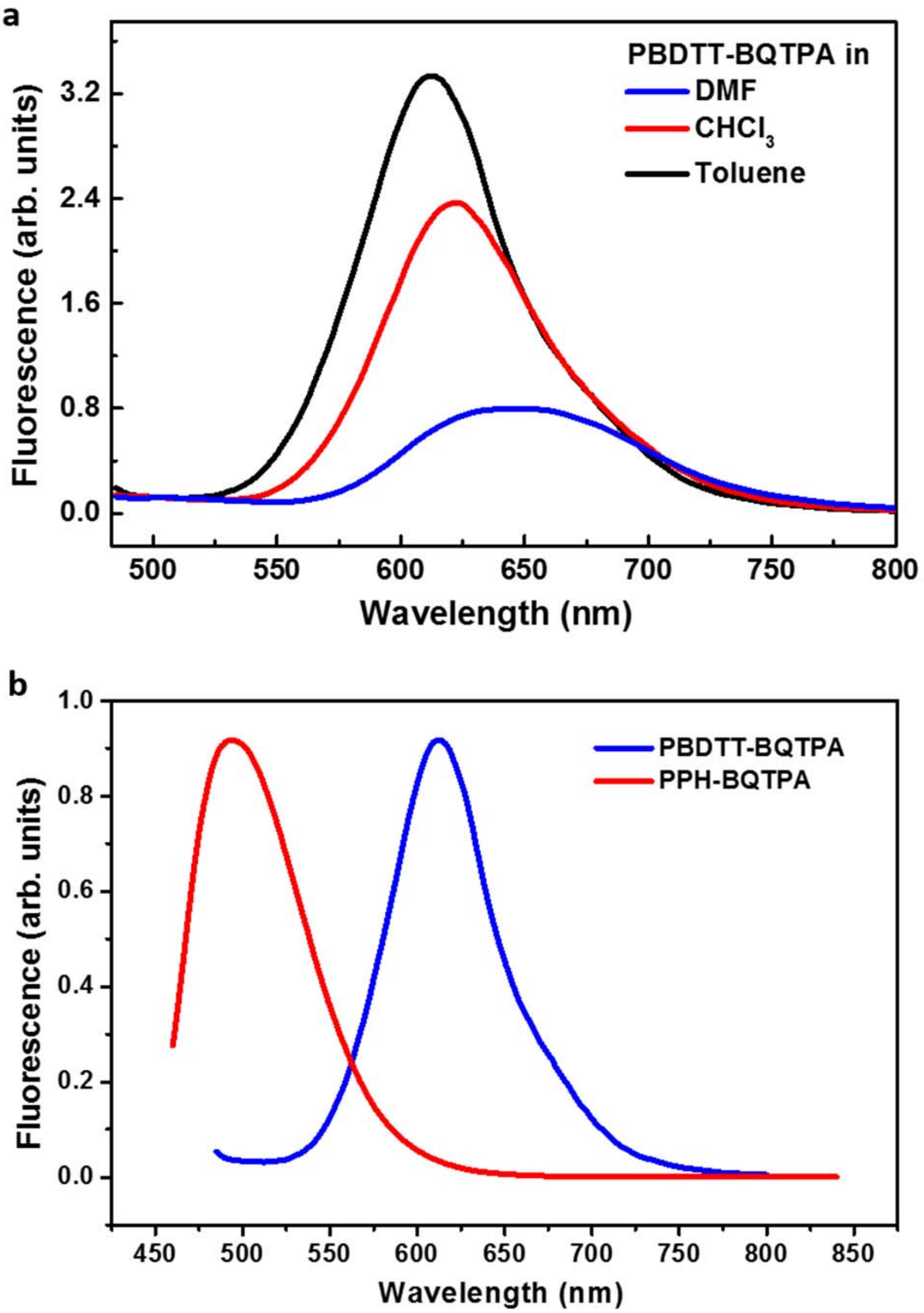

Supplementary Figure 4. Fluorescence spectra. (a) PBDTT-BQTPA in toluene, chloroform and dimethyl formamide (DMF) solutions and (b) PBDTT-BQTPA and PPH-BQTPA in toluene solutions, respectively.

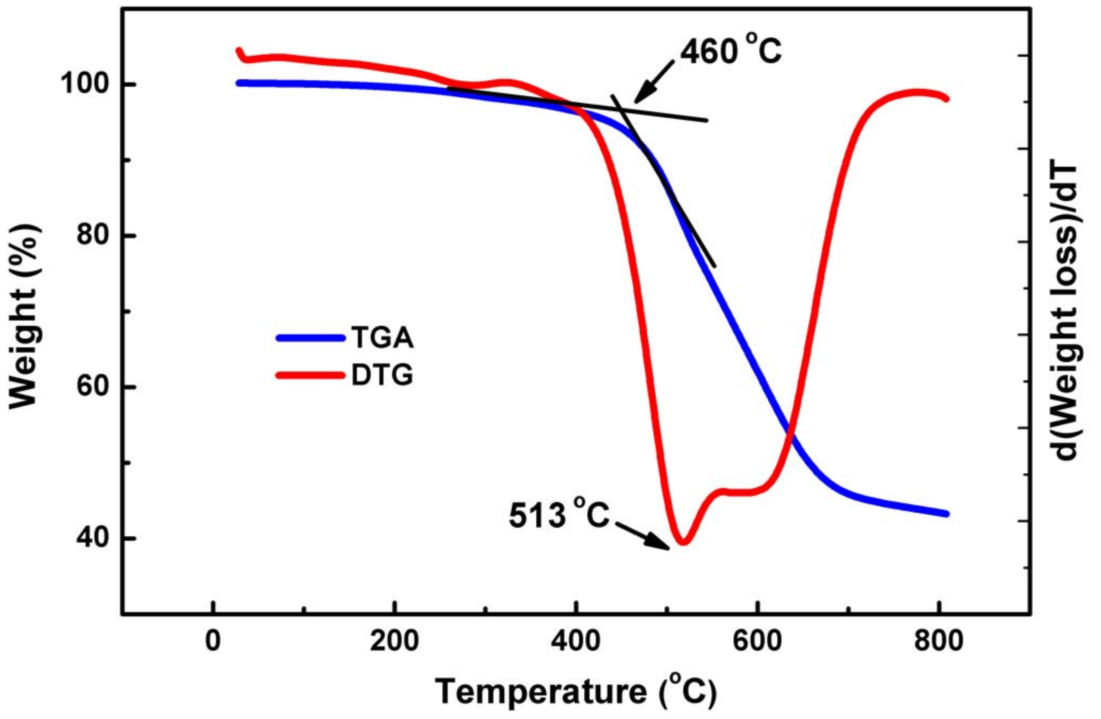

Supplementary Figure 5. Thermal performance of PBDTT-BATP. Thermogravimetry (TG) and differential thermogravimetry analysis (DTG) plots of PBDTT-BQTPA.

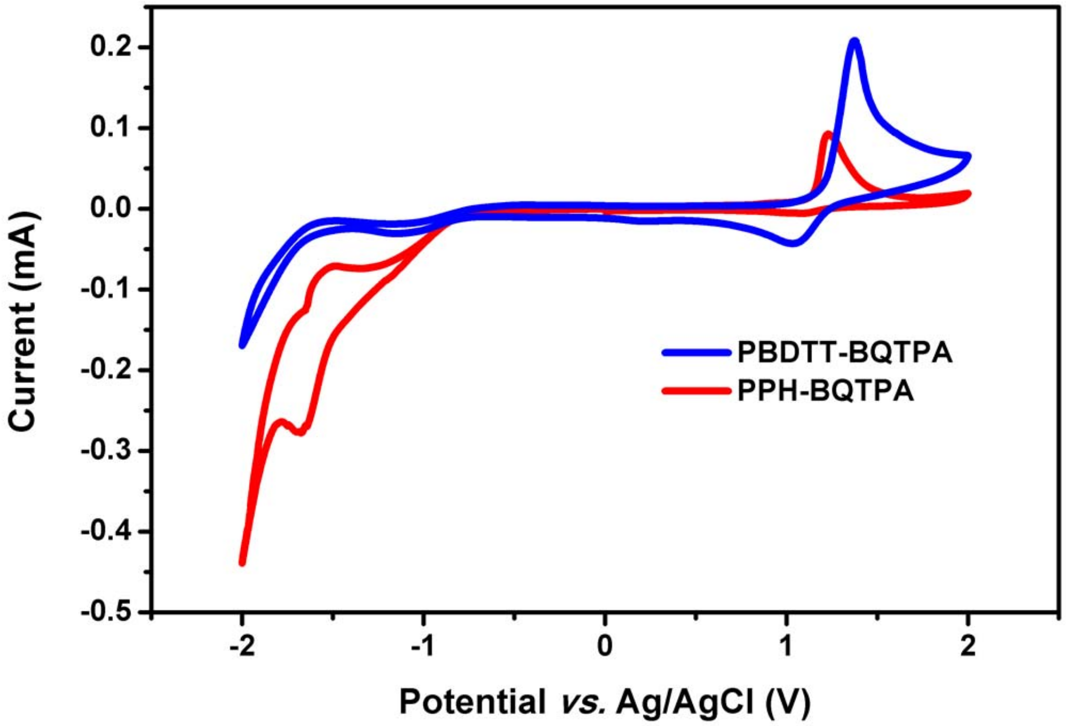

Supplementary Figure 6. Redox behaviors. Cyclic voltammetry plots of PBDTT-BQTPA and PPH-BQTPA.

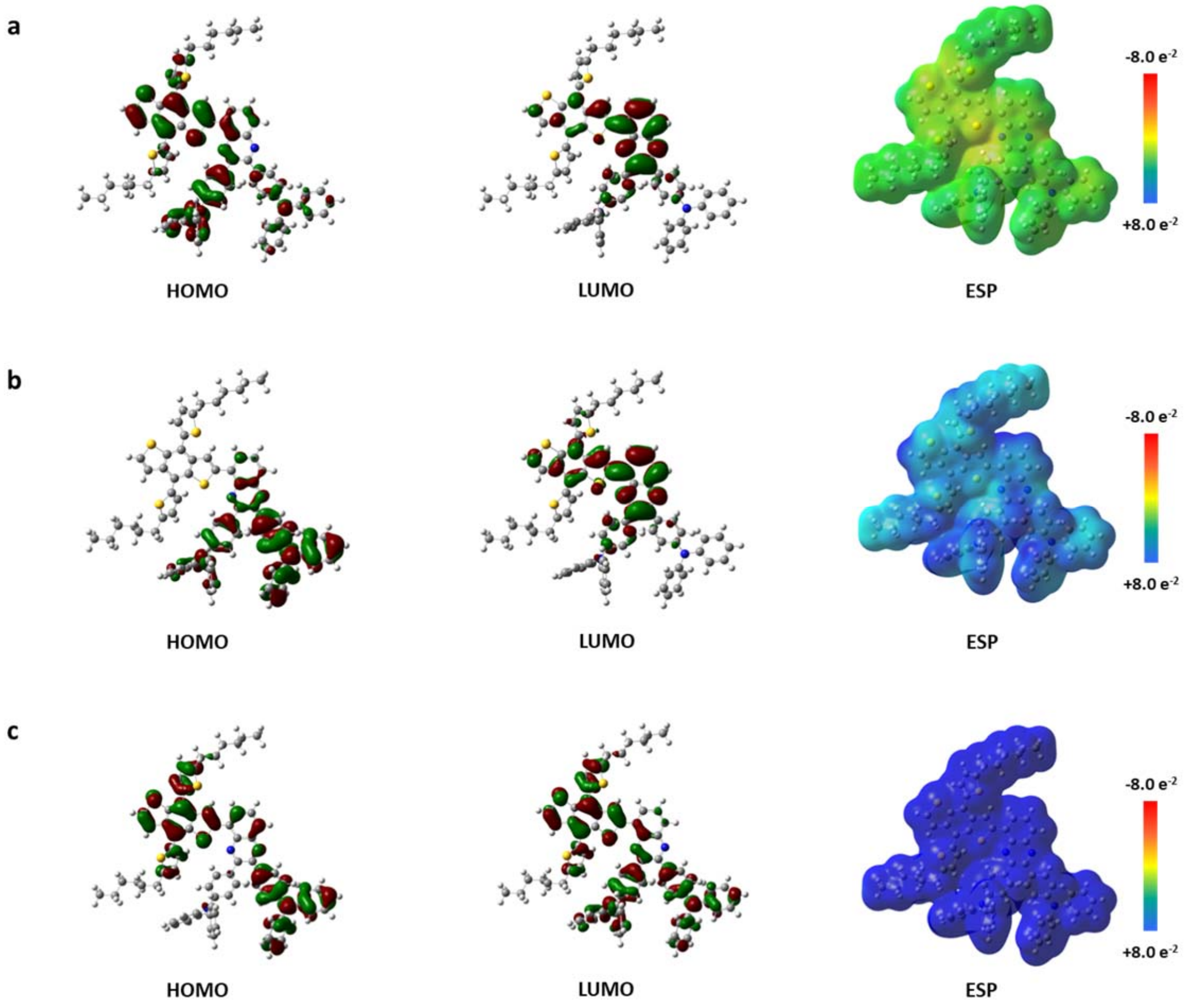

Supplementary Figure 7. Electronic structures of BDTT-BATP. Simulated highest occupied molecular orbitals (HOMO, left panels), lowest unoccupied molecular orbitals (LUMO, middle panels) and electrostatic potential (ESP) surfaces (right panels) of the repeating unit of PBDTT-BQTPA in the (a) initial neutral, (b) +1 oxidized and (c) +2 oxidized states, respectively.

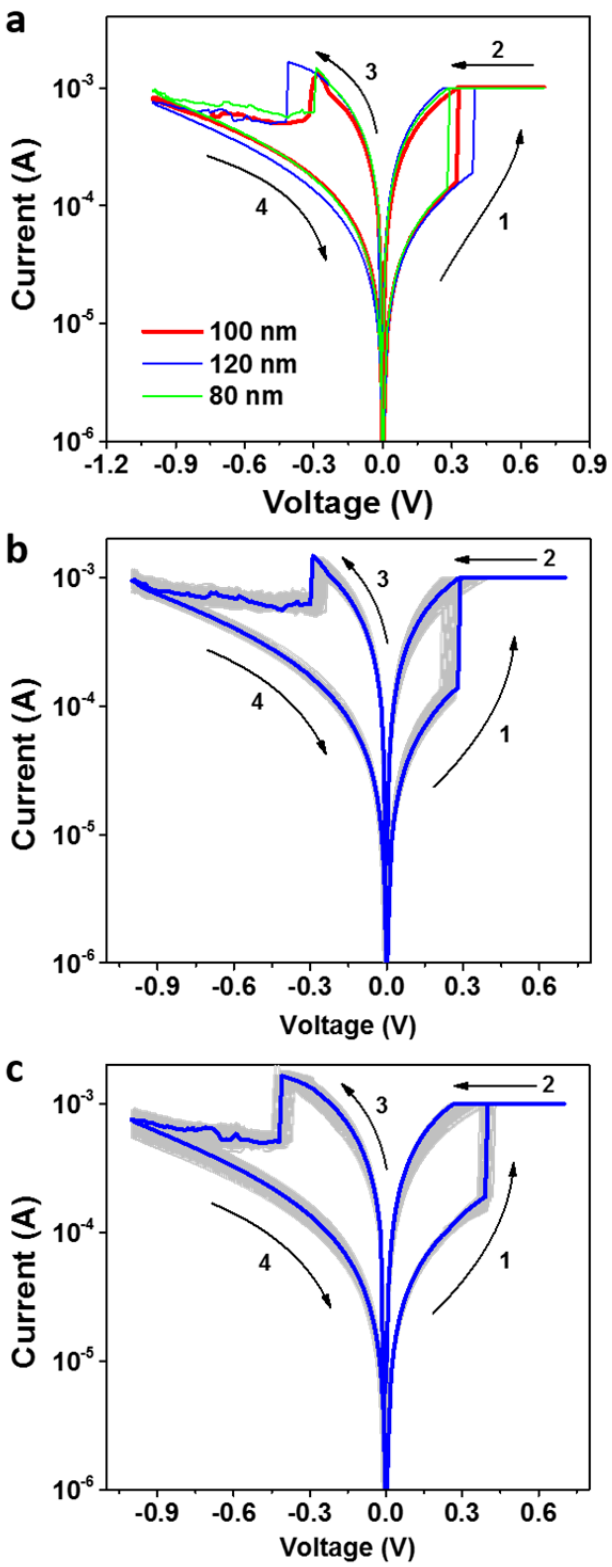

Supplementary Figure 8. Thickness-dependent switching behavior of PBDTT-BATPA. (a) Comparison of the current-voltage curve and resistive switching characteristics of PBDTT-BQTPA memristors with polymer film thickness of 80 nm, 100 nm and 120 nm, respectively. Repeatable current-voltage curves of PBDTT-BQTPA memristors with polymer film thickness of (b) 80 nm and (c) 120 nm, respectively.

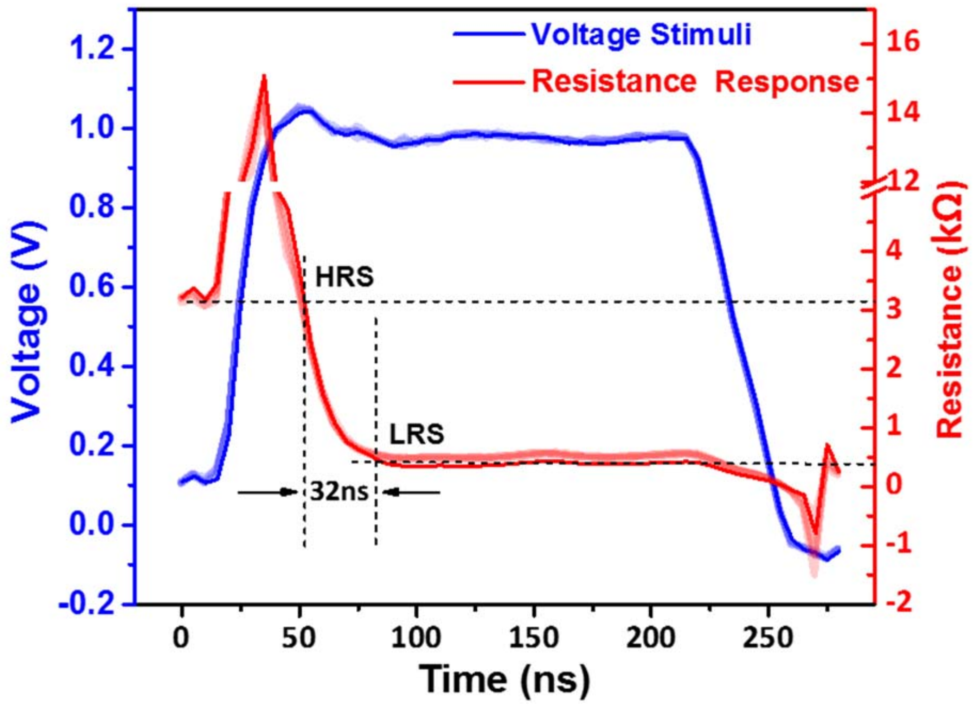

Supplementary Figure 9. Switching speed of PBDTT-BQTPA device. Device resistance in response to the applied voltage stimulus with the amplitude of 1 V and pulse width of 200 ns. The delay-time between when the device resistances reach the respective values of HRS and LRS is estimated as the experimentally recorded switching time of the PBDTT-BQTPA memristor device. A total number of 5 traces of the device resistance responses have been recorded to measure the switching speed, while all the datasets show similar lineshapes.

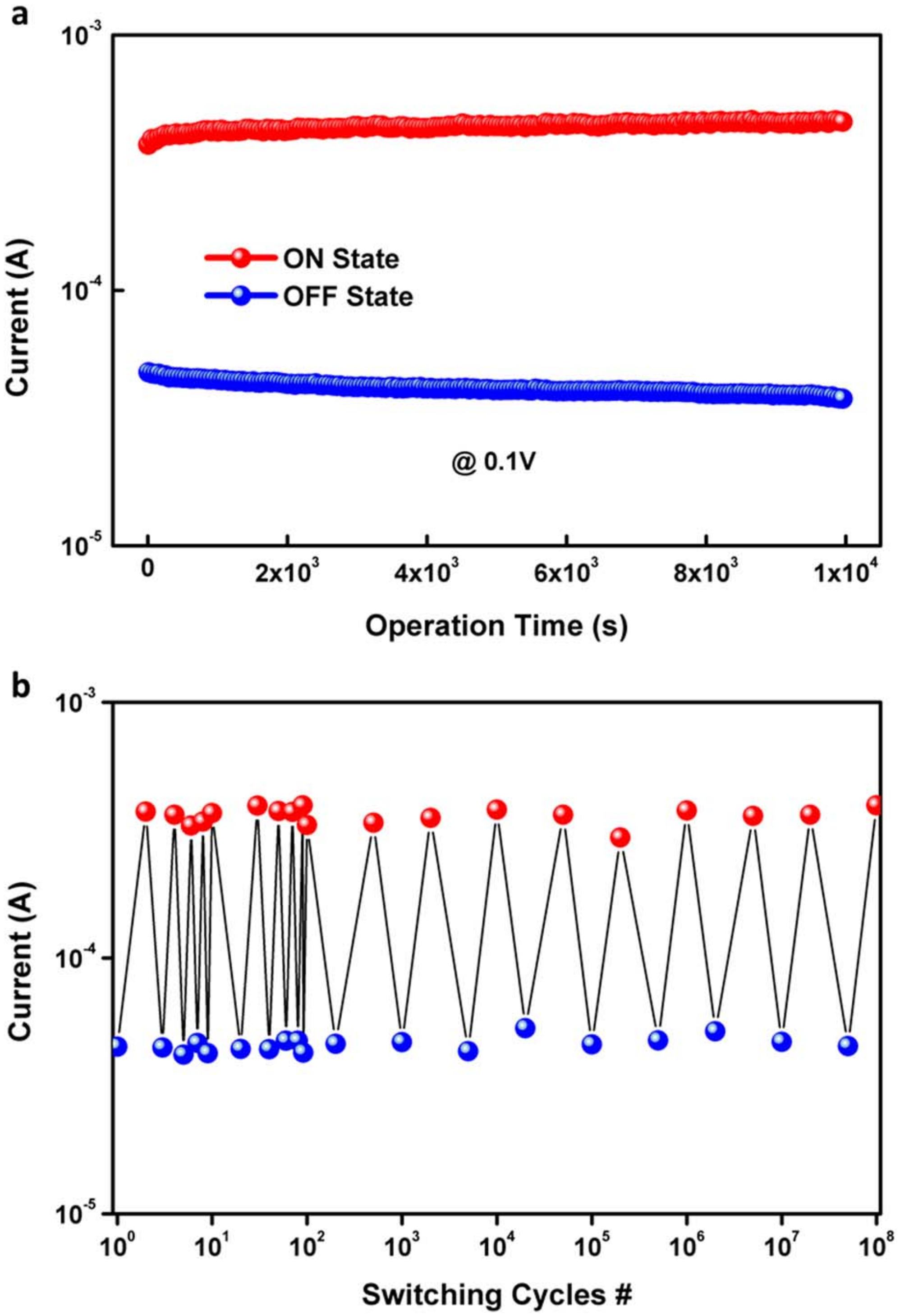

Supplementary Figure 10. Stability and reliability of the PBDTT-BATPA Devices. (a) Retention and (b) endurance characteristics of the PBDTT-BQTPA memristor.

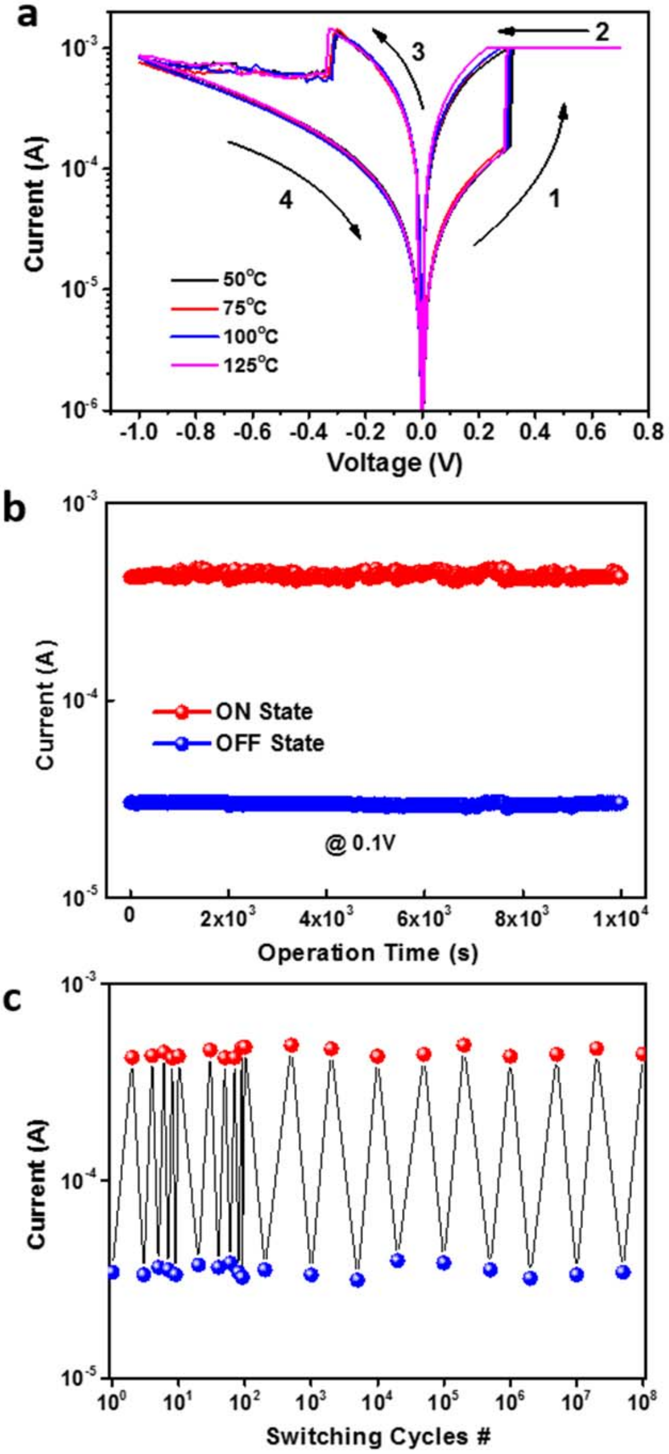

Supplementary Figure 11. Thermal stability of the PBDTT-BATPQ devices. (a) Current-Voltage characteristics of the PBDTT-BQTPA device showing repeatable switching chatacteristics at the elevated temperatures of 50 ^{o}C, 75 ^{o}C, 100 ^{o}C, 125 ^{o}C, respectively. (b) Retention and (c) endurance characteristics of the device at 125 ^{o}C.

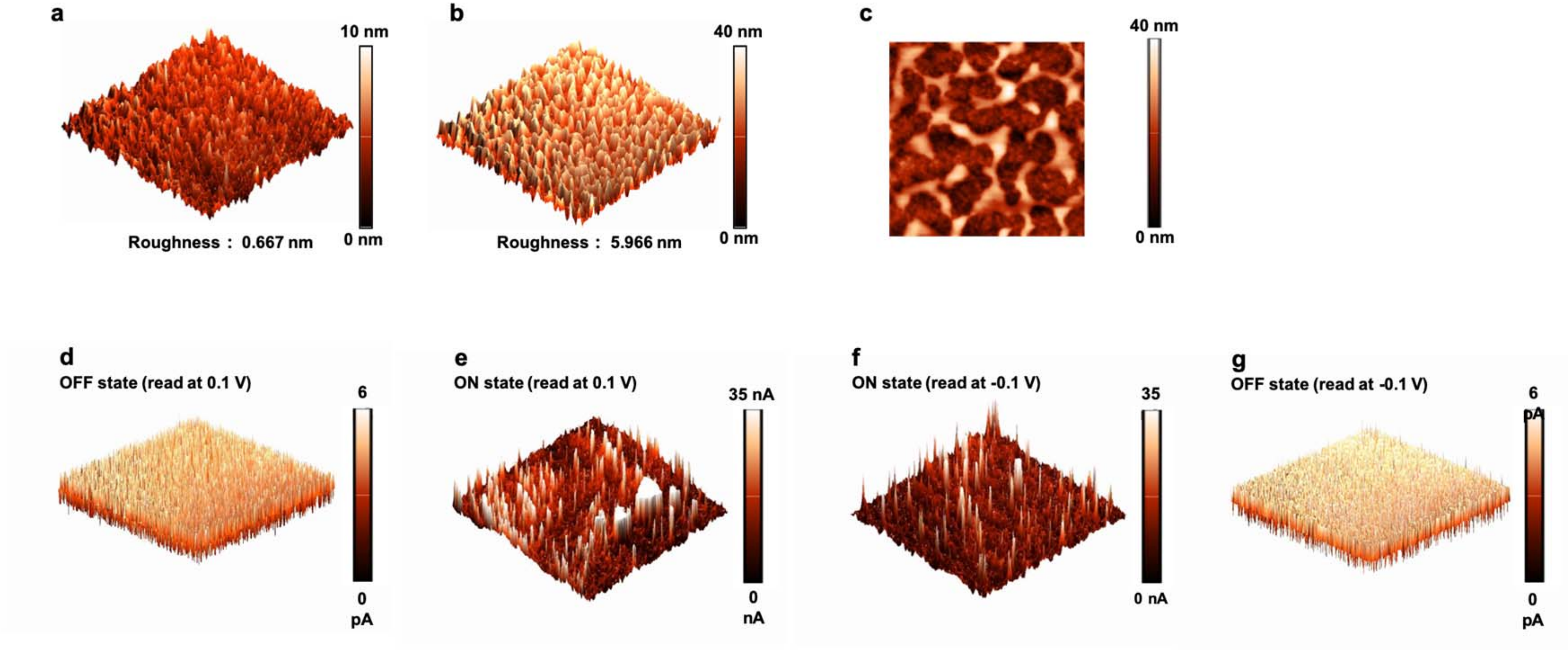

Supplementary Figure 12. Morphology and microscopic switching characteristics. Three-dimensional morphology of (a) PBDTT-BQTPA and (b) PPH-BQTPA thin films over the scanning area of 5 μm × 5 μm. (c) Two-dimensional morphology and (d to g) ON/OFF state current mapping images of the PPH-BQTPA thin film over the scanning area of 5 μm × 5 μm. Localized resistive switching and filamentary conduction behaviors are observed in PPH-BQTPA thin films.

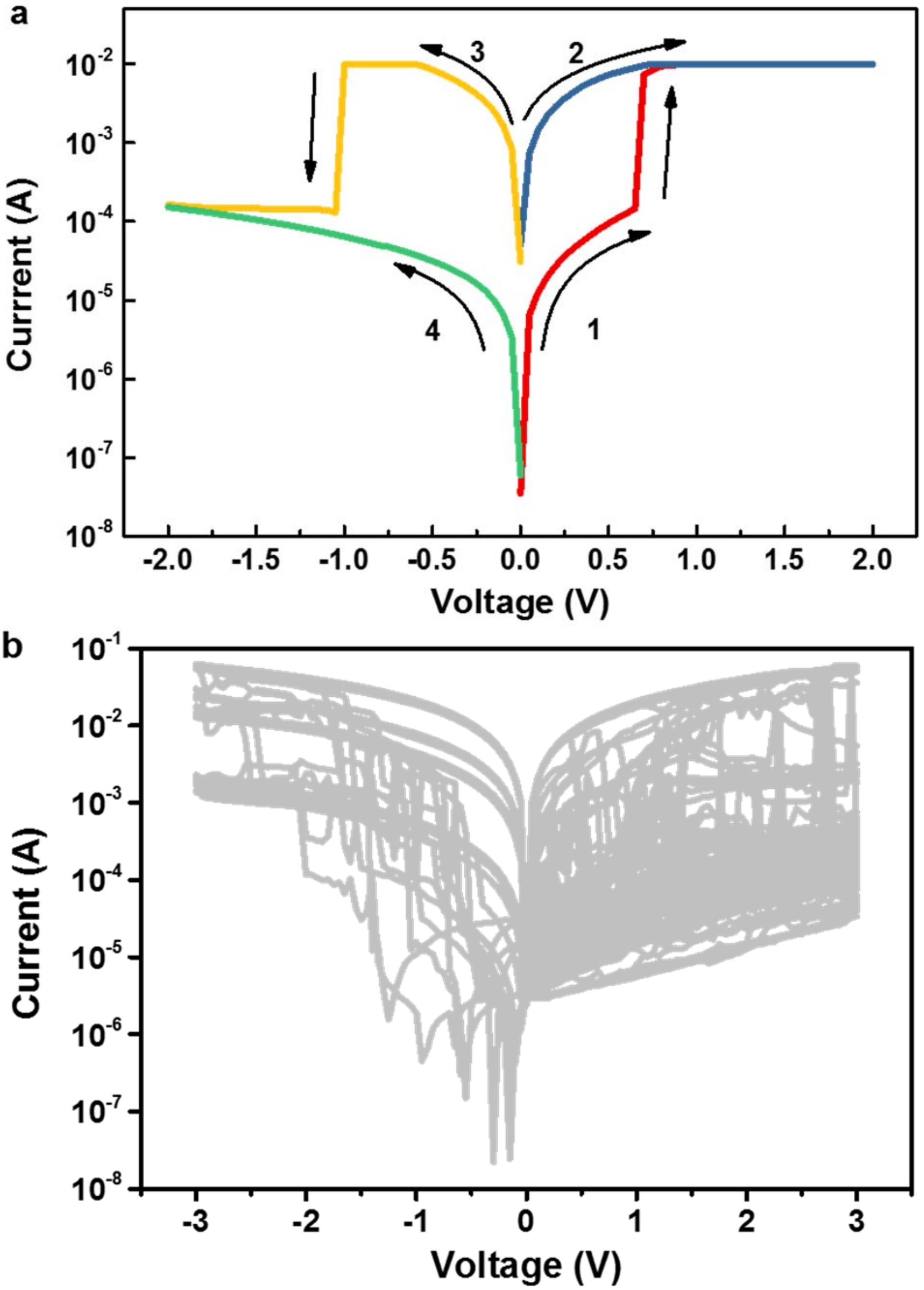

Supplementary Figure 13. Current-voltage characteristics of the Au/PPH-BQTPA/ITO device. (a) nonvolatile bipolar resistive switching behavior and (b) stochastic cycle-to- cycle variations.

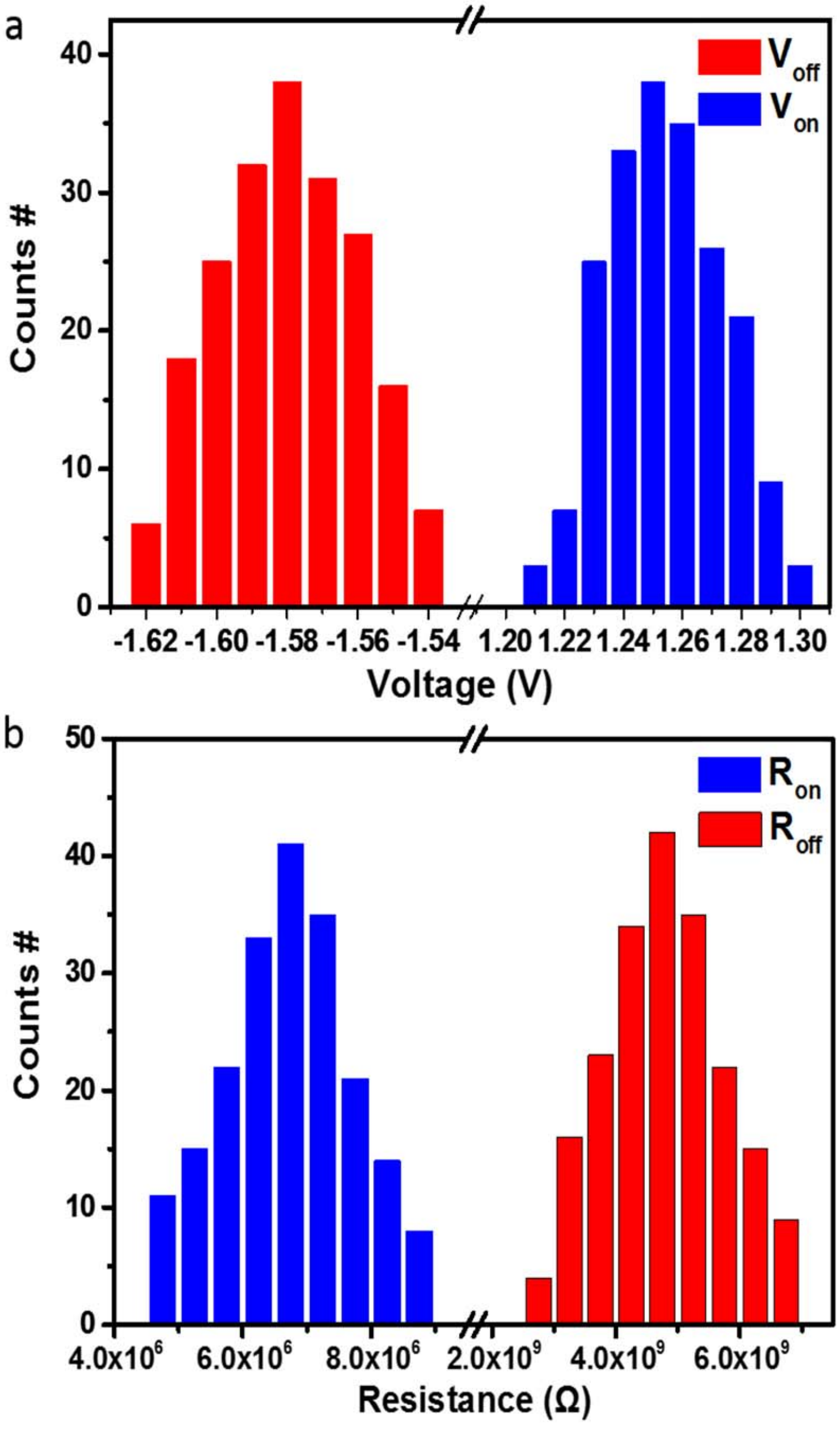

Supplementary Figure 14. Switching uniformity of the nanoscale memristor array. (a) Switching voltages and (b) device resistances distribution of the nanometer scale PBDTT-BQTPA memristor device fabricated in an 8 × 8 crossbar array.

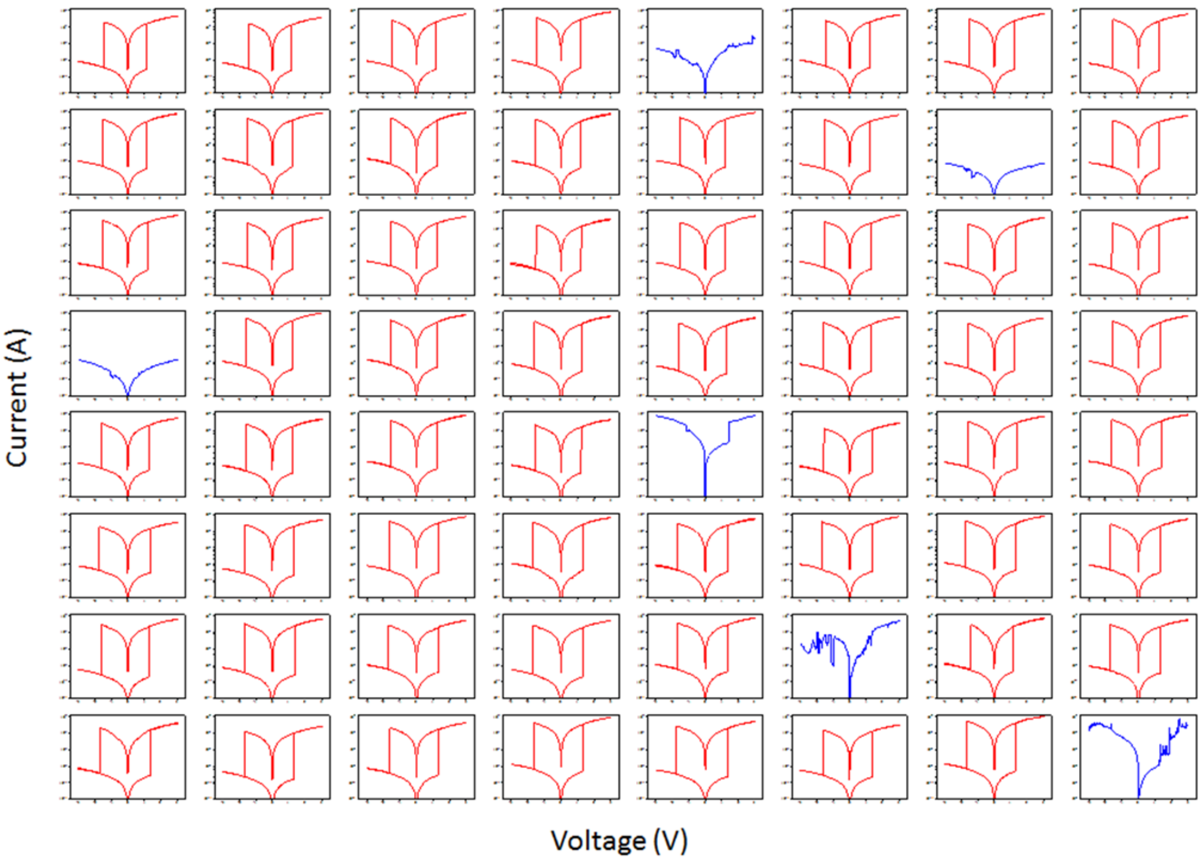

Supplementary Figure 15. Current-voltage characteristics of all the 64 nanometer scale PBDTT-BQTPA memristor devices in an 8 × 8 crossbar array. All the I-V curves were obtained by sweeping the voltage in the sequence of 0 V → 3 V → 0 V → -3 V → 0 V. The current and voltage axes of all the figures above are ranging from 10^{-11} A to 1.3×10^{-6} A, and from -3.5 V to 3.5 V, respectively.

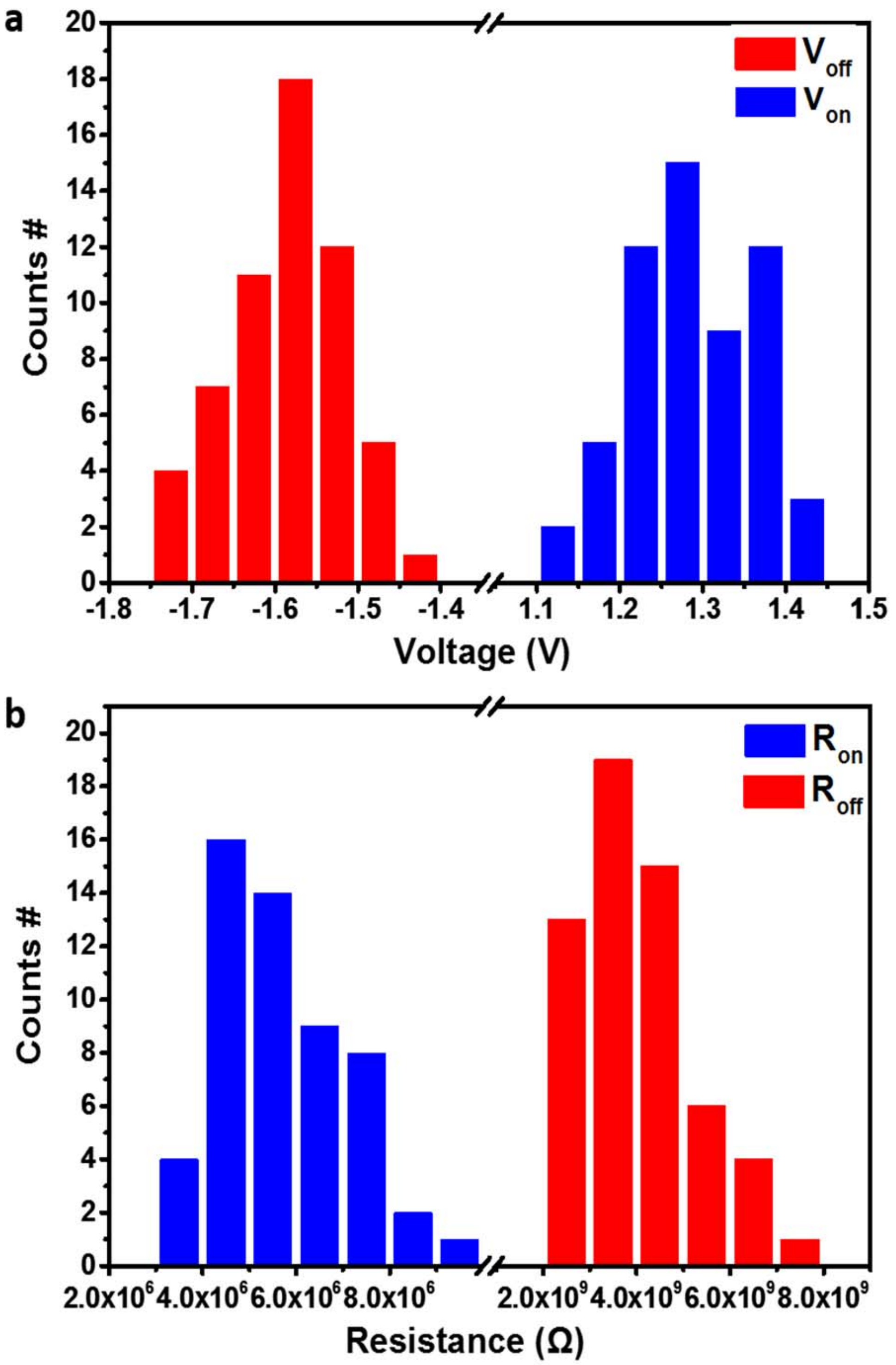

Supplementary Figure 16. Histogram of the switching voltage and ON/OFF ratio of the 64 nanometer scale PBDTT-BQTPA memristor devices in an 8 × 8 crossbar array. (a) Switching voltages and (b) device resistances distribution of the 58 nanometer scale PBDTT-BQTPA memristor devices derived from Supplementary Figure 15.

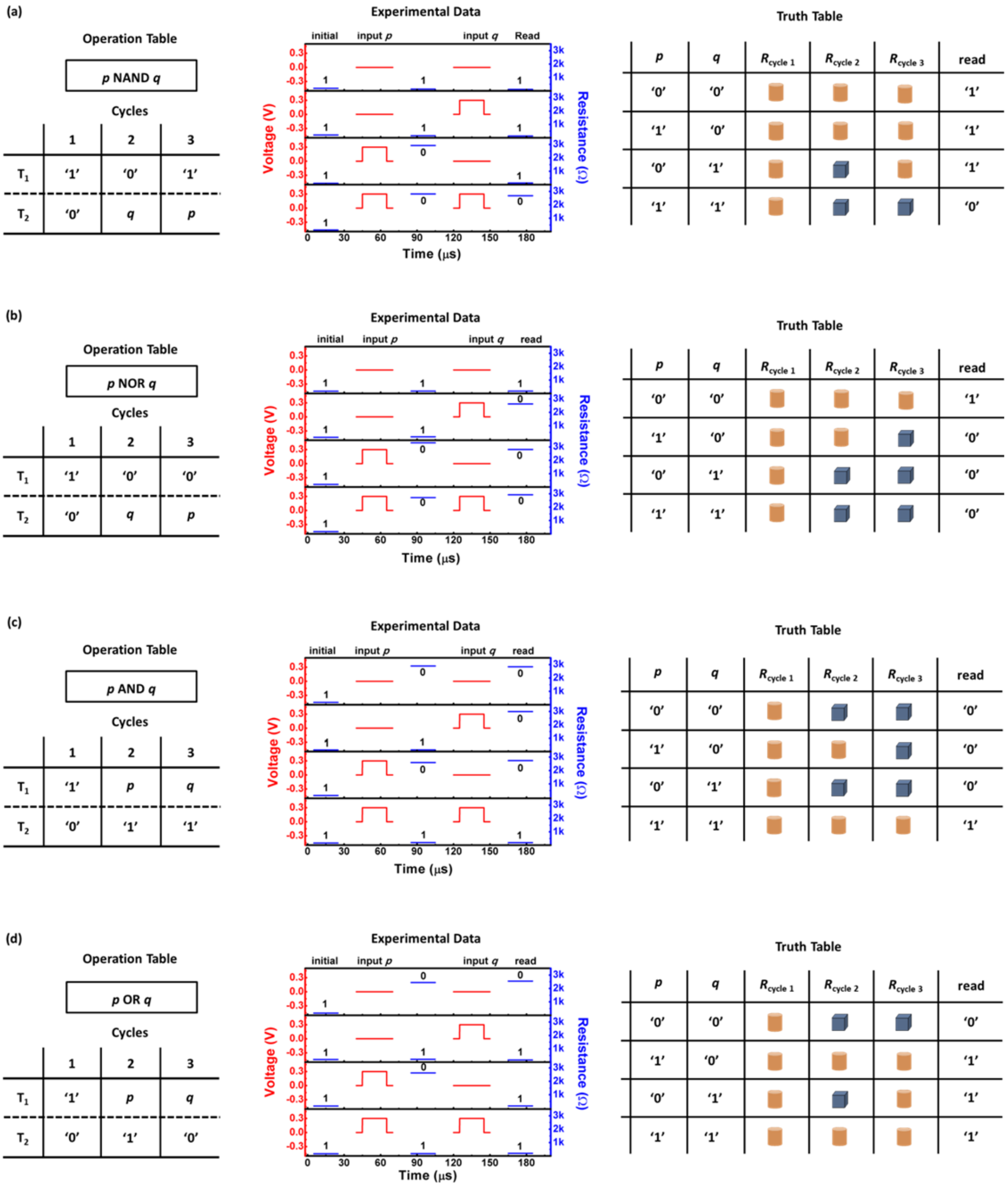

Supplementary Figure 17. Logic computing with PBDTT-BQTPA memristors. Operation methodologies, experimental data sets and truth tables for the (a) NAND, (b) NOR, (c) AND and (d) OR logic gates.

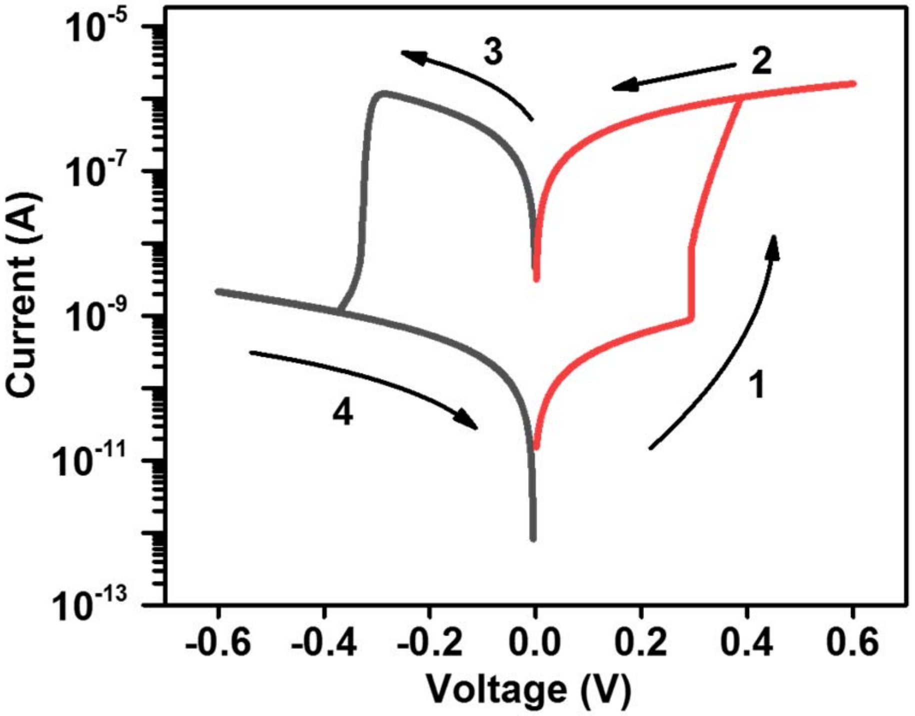

Supplementary Figure 18. Device modeling of the PBDTT-BQTPA memristor. Mathematically modeled current-voltage characteristics of the PBDTT-BTPA memristor device using verilog-A language.

Supplementary Tables

Supplementary Table 1 Truth table and operation methodology of a parallel 1-bit full adder

**Logic Operation**	**Full Adder**								
Input	A	0	0	0	0	1	1	1	1
	B	0	0	1	1	0	0	1	1
	C_{in}	0	1	0	1	0	1	0	1
Output	Sum	0	1	1	0	1	0	0	1
	C_{out}	0	0	0	1	0	1	1	1
Sum & Carry	M_1: V_T=1-0	M_2: V_T=0-1	M_3: V_T=0-1	M_4: V_T=1-0	M_5: V_T=1-0				
	M_1: V_T=1-0	M_2: V_T=A-0	M_3: V_T=A-0	M_4: V_T=0-A	M_5: V_T=0-C_{in}				
	M_1: V_T=B-1	M_2: V_T=0-C_{in}	M_3: V_T=B-1	M4: V_T=B-1	M_5: V_T=1-B				
	M_1: V_T=C_{in}-1	M_2: V_T=0-B	M_3: V_T=C_{in}-1	M_4: V_T=C_{in}-0	M_5: V_T=0-A				

Supplementary Table 2 Sequential operation methodology of the parallel 1-bit full adder

**Cycles**	**BL1**	**BL2**	**BL3**	**BL4**	**WL1**	**WL2**	**WL3**	**Working Devices**	**Output**
1	/	1			/0//			M_1	
2	/	/	0	0		//11		M_2、M_3	
3	1	1					00//	M_4、M_5	
4	/	1	/	/	//0/			M_1	
5	/	/	A	A		//00		M_2、M_3	
6	0	0	/	/			C_{in}A//	M_4、M_5	
7	/	B	/	/	/1//			M_1	
8	/	/	B	0		//1C_{in}		M_2、M_3	
9	1	B	/	/			B1//	M_4、M_5	
10	/	C_{in}	/	/	/$\overline{A}$//			M_1	$A \bullet B + A \bullet C_i + B \bullet C_i$
11	/	/	C_{in}	0		//1B		M_2、M_3	$A \oplus B \oplus C_i$
12	0	C_{in}	/	/			A0//	M_4、M_5	
13	/	V_{read}	/	/	/0//			M_1	Sum
14	V_{read}	V_{read}	V_{read}	V_{read}		//00	00//	M_2、M_3 M_4、M_5	C_{out}

Supplementary Table 3 Operating parameters of the hidden layers

**Convolutional Layer**	**C1**	**C3**
Size of input feature maps	28×28	(12×12) ×20
Size of convolution kernels	5×5	(5×5) ×20
Number of convolution kernels	20	50
Number of output feature maps	20	50
Size of output feature maps	24×24	8×8
Number of trainable parameters	5×5×20+20	(5×5) ×20×50+50
Pooling Layer	S2	S4
Size of input feature maps	(24×24) ×20	(8×8) ×50
Size of pooling filter	2×2	2×2
Number of output subsampling maps	20	50
Size of output subsampling maps	12×12	4×4
Fully-Connected Layer	F5	F6
Size of input feature maps	(4×4) ×50	1×500
Number of output feature maps	1	1
Size of output feature maps	500	10
Number of trainable parameters	4×4×50×500+500	500×10+10

Supplementary Table 4 Number of subarrays for BNN and MNIST image recognition

Layer	Type	Kernel Size	Subarray 64×64
C1	Convolutional	(20, 1, 5, 5)	N/A
C3	Convolutional	(50, 20, 5, 5)	16
F5	Fully-connected	(800, 500)	25×8
F6	Fully-connected	(500, 10)	8
Total	N/A	N/A	224

Supplementary Notes

Supplementary Note 1: Synthesis and Characterization of Monomers and Polymers

All chemicals were purchased from Aldrich and used as received without further purification. Organic solvents were purified, dried, and distilled under dry nitrogen. The compounds **1** and **2** were prepared according to the reported procedures,[1] and 4,8-dehydrobenzo[l,2-b:4,5-b']di thiophene-4,8-dione (**3**) was purchased from Solarmer Materials Inc.

Synthesis of 4,4'-(5,8-dibromoquinoxaline-2,3-diyl)bis(N,N-diphenylaniline) (**M1**): A mixture of compound **1** (0.55 g, 1 mmol), compound **2** (0.27 g, 1 mmol) and acetic acid (20 mL) was heated at 70 °C for 6 h in a nitrogen atmosphere. After cooling to room temperature, the reaction mixture was poured into deionized water (100 mL). The collected precipitate was purified through column chromatography (CH_2Cl_2:hexane = 1:2) to give a yellow solid (0.38 g, yield: 49%). ^{1}H NMR ($CDCl_3$, 400 MHz), δ/ppm: 6.96-7.04 (d, 4H), 7.05-7.13 (m, 4H), 7.14-7.19 (d, 8H), 7.25-7.33 (m, 8H), 7.55-7.64 (d, 4H), 7.82-7.88 (s, 2H).

Synthesis of 4,8-bis(5-hexylthiophen-2-yl)benzo[1,2-b;4,5-b']dithiophene (**5**): Under the protection of argon, n-butyllithium (2.4 M, 18 mL) was added dropwisely to 2-hexylthiophene (**4**) (6.72 g, 40 mmol) in tetrahydrofuran (THF, 60 mL) at 0°C. Then the mixture was warmed up to 50 °C and stirred for 1 h. Subsequently, 4,8-dehydrobenzo[l,2-b:4,5-b']dithiophene-4,8-dione (**3**) (2.2 g, 10 mmol) was added, and the mixture reacted for another 1 h at 50 °C. After cooling down to room temperature, $SnCl_2·2H_2O$ (18 g, 80 mmol) dissolved in 10% HCl (32 mL) was added into the system, and the mixture was stirred for additional 1.5 h before being poured into ice water. Extraction was executed by diethyl ether twice to remove the inorganic

phases, and the combined organic phase was concentrated to obtain raw **5**. Further purification was carried out by column chromatography using petroleum ether as eluent to obtain pure **5** as a light-yellow liquid (2.32g, yield 44.4%). ^{1}H NMR ($CDCl_3$, 400 MHz), δ/ppm: 7.63-7.71 (t, 2H), 7.39-7.42 (m, 2H), 7.27-7.31 (d, 2H), 6.87-6.92 (t, 2H), 2.81-2.93 (m, 4H), 1.72-1.83 (m, 4H), 1.40-1.45 (t, 4H), 1.32-1.35 (d, 8H), 0.9-1.0 (t, 6H).

Synthesis of the monomer bis(trimethyltin)-4,8-bis(5-hexylthiophen-2-yl)benzo[1,2-b:4,5-b']dithiophene (**M2**): A solution of compound **5** (1.05 g, 2 mmol) in THF (40 mL) at 0 °C was placed in a 150 mL argon purged flask, and then n-butyl lithium (2.4 M, 1.8 mL) was added. The reaction mixture was stirred for 2 h at ambient temperature. Subsequently, chlorotrimethylstannane (1.0 M in hexane, 4.8 mL) was added and the mixture was stirred for an additional 1 h at room temperature. Then the mixture was extracted by diethyl ether and the combined organic phase was concentrated to obtain **M2**. Further purification was carried out by recrystallization using ethanol to obtain the pure **M2** as a light-yellow solid (3.22 g, yield 73.2%). ^{1}H NMR ($CDCl_3$, 400 MHz), δ/ppm: 7.61-7.69 (t, 2H), 7.25-7.29 (d, 2H), 6.86-6.90 (t, 2H), 2.76-2.91 (m, 4H), 1.68-1.79 (m, 4H), 1.38-1.41 (t, 4H), 1.29-1.34 (d, 8H), 0.87-0.96 (t, 6H), 0.23-0.68 (m, 18H).

Synthesis of the 2D conjugated polymer **PBDTT-BQTPA**: Equimolecular amounts of monomer **M1** (387 mg, 0.5 mmol) and monomer **M2** (425 mg, 0.5 mmol), and anhydrous toluene (10 mL) were added to a 25 mL Schlenk flask. The reaction mixture was bubbled with argon for 15 min, and then 20 mg of $Pd(PPh_3)_4$ was added. The reaction mixture was vigorously stirred at 110 °C in an argon atmosphere for 24 h. The collected precipitate from meth-

anol was further put into Soxhlet apparatus to remove the oligomers and catalyst residues for 24 h. The product was extracted by THF for another 24 h. The THF extracts were concentrated and the product was precipitated by methanol, collected by filtration and dried under vacuum overnight. Finally, the 2D conjugated polymer **PBDTT-BQTPA** of 504 mg (yield 89%) was obtained. GPC (THF as eluent, **Supplementary Figure 2a**): Mn = 1.29×10^4, PDI = 1.46. ^{1}H NMR ($CDCl_3$, 400 MHz), δ/ppm: 8.47-8.61 (d, 4H), 8.12-8.24 (d, 2H), 7.61-7.73 (m, 4H), 7.02-7.31 (m, 22H), 6.82-7.00 (m, 4H), 2.80 (t, 4H), 1.65-1.80 (m, 4H), 1.13-1.37 (m, 12H), 0.70-0.90 (m, 6H).

Synthesis of the polymer **PPh-BQTPA**: **PPh-BQTPA** was prepared through Suzuki reaction. 1,4-bis(4,4,5,5-tetramethyl-1,3,2-dioxaborolan-2-yl)benzene (115 mg,0.5 mmol), **M1** (387.2 mg, 0.5 mmol), tetrakis(triphenylphosphine)palladium (12 mg) along with potassium carbonate (272 mg, 2 mmol) were added into a 25ml flask in Ar atmosphere. Degassed toluene (10 mL) and deionized water (2 mL) was injected into the mixture by syringe. Then the reactant was stirred at 80 °C in Ar atmosphere for 48h. The mixture was poured into 200 mL methanol for precipitation. Soxhlet extraction with acetone took place following the filtration. The obtained polymer was then dried under vacuum at 50 °C overnight to give 313.2 mg (yield: 87%) yellowish solid. GPC (THF as eluent, **Supplementary Figure 2b**): Mn = 1.19×10^4, PDI = 1.31. ^{1}H NMR ($CDCl_3$, 400 MHz), δ/ppm): 7.84 (s, 2H), 7.57-7.63 (d, 4H), 7.29-7.36 (m, 10H), 7.12-7.19 (d, 8H), 7.06-7.11 (t, 6H), 6.97-7.05 (d,4H).

Supplementary Note 2: UV-Visible Absorption Spectra of Polymers

The UV-Visible absorption spectra of PBDTT-BQTPA and the control sample PPH-BQTPA without 2D donor moiety of BDTT in the repeating unit, recorded in solvents of different polarities, are shown in **Supplementary Figure 3**. The absorption maximum of the PBDTT-BQ TPA toluene solution appears at the wavelength of 332 nm, which is accompanied by a pair of shoulder peaks at 310 nm and 370 nm, respectively (**Supplementary Figure 3a**). A moderate broad absorption band in the wavelength range of 460 nm - 630 nm, with its apex located at 478 nm, is also observed. With the increasing of the solvent polarity from toluene to chloroform ($CHCl_3$) and dimethyl formamide (DMF), these four peaks move to 310 nm, 332 nm, 370 nm, 489 nm in $CHCl_3$ and 306 nm, 333 nm, 364 nm, 489 nm in DMF, respectively. The blue shift of the major absorption peaks from 332 nm and 478 nm to 333 nm and 489 nm suggests that these two peaks are arising from the n-π* transition of the thiophene and quinoxaline units, while the red shift of the 310 nm and 370 nm absorption shoulders to 306 nm and 364 nm indicates that they can be assigned to π-π* transition of the conjugated polymer backbone.

Supplementary Note 3: Electrochemical Analysis of PBDTT-BQTPA and PPH-BQTPA

Cyclic voltammetry measurements have been performed to investigate the electrochemical redox activity of the 2D conjugated polymer PBDTT-BQTPA and control sample PPH-BQ TPA. The 2D conjugated polymer thin film coated on a Pt disk electrode was scanned in 0.1 M acetonitrile solution of Bu_4NPF_6, with Ag/AgCl and a platinum wire as the reference and counter electrode, respectively. As shown in **Supplementary Figure 6**, PBDT-BQTPA exhibits a redox behavior, with an onset oxidation potential of 1.13 V and an onset reduction potential of -0.76 V. According to the equation: HOMO/LUMO=-[$E_{ox/red}$ - $E_{ox(ferrocene)}$] - 4.8, where E_{ox} is the onset oxidation potential, E_{red} is the onset reduction potential, and $E_{ox(ferrocene)}$ is the onset oxidation potential of ferrocene (0.39 V versus Ag/AgCl),[2] the HOMO and LUMO energy levels of PBDT-BQTPA are estimated to be -5.54 and -3.65 eV, respectively. When PBDTT-BQTPA is sandwiched between the Au top electrode and ITO bottom electrode, the energy barrier for hole injection from the electrodes into the polymer will be 0.44 eV (which is the energy difference between the work function of Au, -5.1 eV, and the HOMO level of the polymer, -5.54 eV), while the energy barrier for electron injection will be 1.15 eV (energy difference between the work function of ITO, -4.8 eV, and the LUMO level of the polymer, -3.65 eV). Thus, hole injection and transport will play a dominated role in the Au/PBDT-BQTPA/ITO device. Similarly, the control sample PPH-BQTPA shows onset oxidation and reduction potentials of 1.17 V and -0.84 V, giving rise to HOMO, LUMO and energy band gap of -5.58 eV, -3.57 eV, and 2.01 eV, respectively.

Supplementary Note 4: Molecular Simulation of PBDTT-BQTPA

To give insight into the electronic structure of the 2D conjugated polymer, the molecular electrostatic potential (ESP) surfaces, highest occupied molecular orbitals (HOMO) and lowest unoccupied molecular orbitals (LUMO) of the basic unit of BDTT-BQTPA were simulated by density functional theory (DFT) in the B3LYP/6-31G(d) level with the Gaussian 09 program package. As shown in the **Figure 4f**, due to the extended π-conjugation over two-dimensional molecular structure and the ground state charge transfer interaction occurred between the 2D thiophene-quoinxaline D-A pair, continuous positive ESP regions can be observed throughout the conjugated backbones as well as the conjugated side chains of BDTT-BQTPA. In the neutral state, the electron clouds on HOMO are mainly focused on the 2D thiophene backbone and triphenylamine moieties, while majority of the electron clouds on LUMO are located on the quinoxaline unitity (**Supplementary Figure 7a**). The calculated HOMO and LUMO energy levels are -4.903 eV and -2.054 eV, respectively, and the deviation from the experimental data can be ascribed to the use of repeating unit during simulation that lacks the considering of π conjugation along the extended polymer backbone. The dipole moment of the neutral ground state BDTT-BQTPA unit is 2.6916 Debye. When one of the redox-active triphenylamine pendants is oxidized (corresponding to +1 state of the repeating unit), HOMO level distribution of the electron clouds shifts from the 2D thiophene backbone to the TPA side-chains obviously (**Supplementary Figure 7b**). The ESP of the BDTT-BQTPA unit also becomes more positive with blue color, giving rise to a greatly increased dipole moment of 5.3743 Debye. Fully oxidization of both TPA groups to +2 state results in a much lower energy bandgap of the polymer, ~ 0.294 eV (**Supplementary Figure 7c**), which make the polymer thin film

highly conductive and accounts for the observed resistive switching characteristics of the Au/PBDTT- BQTPA/ITO memristor devices. The ESP surface turns to darker blue, and the charged repeating unit exhibits an even larger dipole moment of 8.482 Debye. It should be pointed out that during molecular simulation we didn't introduce any counter ion to balance the charge of the oxidized BDTT-BQTPA^{+}/BDTT-BQTPA^{2+} molecules in the vacuum environment, thus the absolute values of the HOMO and LUMO energy levels would be different from those of the actual values. Nevertheless, the lowered energy bandgap of PBDTT-BQTPA will result in device transition the initial HRS into a LRS state.

Supplementary Note 5: In-memory Logic Computing with PBDTT-BQTPA Memristors

In the Au/PBDTT-BQTPA/ITO structured memristor devices, the Au top electrodes and the ITO bottom electrodes are defined as T1 and T2 terminals, respectively. The pulse voltages applied onto either T1 or T2 serve as the logic input signals, while the value of V_{T1}-V_{T2} determines the devices' states during resistive switching and logic operations. V_0=0 V and V_1=0.3 V, with the pulse width of 20 μs, are used as logic inputs "0" and "1" in the present study, while the OFF and ON states with the resistances of 100 Ω~200 Ω and 2.5 kΩ~3.5 kΩ are taken as the logic outputs of "0" and "1", respectively. The output logic (final resistance) state of the memristor can be readout by an additional independent read step with a small voltage pulse of 0.01 V (20 μs pulse width), and is stored directly in the same device non-volatilely.

Supplementary Figure 12 shows the experimental implementation of NAND, NOR, AND and OR operations, respectively. For the NAND operator, the PBDTT-BQTPA device is initially programmed to logic "1" with the resistance of 195 Ω in cycle 1, by setting the T1 and T2 inputs as "1" and "0", respectively. In cycle 2, the Au top electrode T1 is fixed as logic "0", and input *q* is applied onto the ITO bottom electrode T2. In cycle 3, T1 is fixed as logic "1" and input *p* is applied onto T2 (left panel of **Supplementary Figure 13a**). For the *p* and *q* input groups of "0, 0", "1, 0", "0, 1" and "1, 1", the final resistances of the polymer memristor are 114 Ω, 140 Ω, 127 Ω and 2678 Ω, respectively (middle panel of **Supplementary Figure 13a**). Being consistent with the truth table summarized in right panel of **Supplementary Figure 13a**, we confirm that the NAND function is implemented through these three-step operations.

For the NOR operation, the polymer memristor is first initialized to the logic state "1" with the resistance of 170 Ω in cycle 1, by setting the T1 and T2 inputs as "1" and "0", respectively. In the subsequent cycles 2 and 3, the Au top electrode T1 is always fixed as logic "0", while the inputs *q* and *p* are applied onto the ITO bottom electrode T2, respectively (left panel of **Supplementary Figure 13b**). For the *p* and *q* input groups of "0, 0", "1, 0", "0, 1" and "1, 1", the corresponding final resistances of the PBDTT-BQTPA device are 180 Ω, 2624 Ω, 2812 Ω and 2923 Ω (middle panel of **Supplementary Figure 13b**). The experimental data sets are in good agreement with the truth table of the NOR function shown in the right panel of **Supplementary Figure 13b**.

For the AND function, the polymer memristor is initially programmed to logic "1" with the resistance of 180 Ω in cycle 1, during which the T1 and T2 inputs are taken as "1" and "0", respectively. In the subsequent cycles 2 and 3, the ITO bottom electrode T2 is always fixed as logic "1", while the inputs *p* and *q* are applied onto the Au top electrode T1, respectively (left panel of **Supplementary Figure 13c**). For the *p* and *q* input groups of "0, 0", "1, 0", "0, 1" and "1, 1", the corresponding final resistances of the PBDTT-BQTPA device are 2838 Ω, 2992 Ω, 2744 Ω and 191 Ω (middle panel of **Supplementary Figure 13c**). The experimental data sets are in good agreement with the truth table shown in the right panel of **Supplementary Figure 13c**, and confirm that the AND operations have been implemented.

For the OR operator, the PBDTT-BQTPA device is first initialized to logic state "1" with the resistance of 166 Ω in cycle 1, during which the T1 and T2 inputs are taken as "1" and "0", respectively. In cycle 2, the ITO bottom electrode T2 is fixed as logic "1", and input *p* is ap-

plied onto the Au top m electrode T1. In cycle 3, T2 is fixed as logic "0" and input *q* is applied onto T1 (left panel of **Supplementary Figure 13d**). For the *p* and *q* input groups of "0, 0", "1, 0", "0, 1" and "1, 1", the final resistances of the polymer memristor are 2548 Ω, 166 Ω, 162 Ω and 196 Ω, respectively (middle panel of **Supplementary Figure 13d**). These logic outputs are consistent with those shown in the truth table of the OR function, as summarized in right panel of **Supplementary Figure 13d**. The other logic functions of TRUE, FALSE, COPY *p*, NOT *p*, COPY *q*, NOT *q*, IMP, NIMP, RIMP and RNIMP can be implemented similarly within a single device of the present PBDTT-BQTPA memristor, as report in the literatures,[3,4] while the remaining XOR and XNOR has to be achieved by at least involving a pair of the anti-serially connected polymer devices.[5-7] Since the general implementation methodology of these Boolean logic operations are well documented in the references, we herein only show the experimental demonstration of the NAND, NOR, AND and OR gates that are utilized to construct the full adder circuits displayed in **Figure 6**, and skip the others for the avoidance of the necessary redundancies.

Supplementary Note 6: Modeling of the Polymer Memristor and Construction of Memristive Array

The polymer memristive device was mathematically modeled using verilog-A language.[8] The resistance value is constrained by the length and width of its corresponding conductive filament (CF), both of which are functions of the applied voltage and stressing time. The electrical properties of the devices were evaluated using Cadence spectre and the simulation is shown in **Supplementary Figure 18**. Then a bit-cell consisting of the present polymer memristor device and a 180 nm N-metal-oxide-semiconductor (NMOS) transistor from the Semiconductor Manufacturing International Corporation (SMIC) was constructed, after which a 64×64 memristive array was able to be built on the basis of the bit-cells. Note that in order to be electrically compatible with the NMOS transistor obtained from SMIC, the device current of the 400×400 μm^2 PBDTT-BATPA memristor is proportionally reduced to that of a 400×400 nm^2 device according to **Figure 3k**.

Supplementary Note 7: Binary MAC (XNOR) Operation in Memristive Arrays

In binary neural networks, the Multiply-and-Accumulate (MAC) operation can be reduced to XNOR operation.[9] Both weights and input features are constrained to +1 or -1 for convenience in our cases. The weights of a binarized layer in LetNet-5 are stored in memristive arrays, while the input features should be applied sequentially. Each 1-bit XNOR can be accomplished using two bit-cells, wherein the two polymer devices store the weight and the states of the NMOS transistors' gates represent the input features, as depicted in **Figure 6b**. We assume a value of "+1" is stored when the upper polymer (M_u) device is in LRS while the lower (M_l) one is in HRS. On the contrary, a "-1" is stored. Similarly, a "+1" will be input if the upper transistors' gate is open and the lower is close, vice versa. The XNOR result can be represented by the output current, with low current representing "+1" and high current standing for "-1". Subsequently, the accumulated current across the long bit-line is to be used as the summation of all the XNOR results according to the Kirchhoff's current law (KCL). Finally, an analog-to-digital converter (ADC) is used to convert the accumulated current to digital signals.

Supplementary References

1. Albano, G., et al. Chiroptical response inversion upon sample flipping in thin films of a chiral benzo[1,2-b:4,5-b′]dithiophene-based oligothiophene. *Mater. Chem. Front.* **1**, 2047-2056 (2017).
2. Li, D., et al. In-situ growing D-A polymer from the surface of reduced graphene oxide: Synthesis and nonvolatile ternary memory effect. *Carbon* **143**, 851-858 (2019).
3. Borghetti, J., et al. Memristive Switches Enable 'Stateful' Logic Operations *via* Material Implication. *Nature*, **464**, 873-876 (2010).
4. Linn, E., Rosezin, R., Tappertzhofen, S., Böttger, U. & Waser, R. Beyond von Neumann-Logic Operations in Passive Crossbar Arrays alongside Memory Operations. *Nanotechnology* **23**, 305205 (2012).
5. Li, Y., et al. 16 Boolean Logics in Three Steps with Two Anti-Serially Connected Memristors. *Appl. Phys. Lett.* **106**, 233502 (2015).
6. Vourkas, I. & Sirakoulis, G. Ch. Emerging Memristor-Based Logic Circuit Design Approaches: A Review. *IEEE Circuits Sys. Mag.* 3rd Quarter Issue, 15-30, (2016).
7. Gao, S., et al. Improving Unipolar Resistive Switching Uniformity with Cone-shaped Conducting Filaments and Its Logic-in-Memory Application. *ACS Appl. Mater. Interfaces* **10**, 6453-6462 (2018).
8. FitzPatrick, D. & Miller, I. Analog behavioral modeling with the Verilog-A language. Springer Science & Business Media (1998).
9. Courbariaux, M., Hubaea, I., Soudry, D., El-Yaniv, R. & Bengio, Y.. Binarized Neural Networks: Training Deep Neural Network with Weights and Activations Constrained to +1 or -1. arXiv preprint, arXiv: 1602.02830 (2016).
